# Controlling Magnetism in the 2D van der Waals Antiferromagnet CrPS_4_ via Ion Intercalation

**DOI:** 10.1021/acs.nanolett.5c05445

**Published:** 2026-02-10

**Authors:** Alberto M. Ruiz, Diego López-Alcalá, Gonzalo Rivero-Carracedo, Andrei Shumilin, José J. Baldoví

**Affiliations:** † Instituto de Ciencia Molecular, 201469 Universitat de València, Catedrático José Beltrán 2, 46980 Paterna, Spain

**Keywords:** magnetism, intercalation, 2D materials, first-principles calculations, magnonics

## Abstract

Two-dimensional (2D) van der Waals (vdW) magnetic materials are platforms in which inserting chemical species into their interlayer gaps offers a powerful route to engineer magnetism. Here, we focus on the A-type antiferromagnetic semiconductor CrPS_4_ (*T*_N_ = 38 K) and investigate its electronic and magnetic properties upon intercalation of lithium (Li^+^) and organic tetrabutylammonium (TBA^+^) ions using first-principles calculations. Li^+^ incorporation induces a semiconductor-to-metal transition in CrPS_4_ and triggers a switching from an out-of-plane antiferromagnetism state to an in-plane ferromagnetic state. This is accompanied by an increase of the ordering temperature, reaching a 5-fold enhancement for Li_0.5_CrPS_4_. TBA^+^ intercalation expands the vdW gap, decoupling CrPS_4_ layers and stabilizing in-plane ferromagnetism with *T*_C_ > 100 K. Furthermore, it enhances magnon group velocities and yields more isotropic magnon transport. This work highlights intercalation as a powerful approach for tailoring magnetism, paving the way for tunable 2D-layered magnetic materials for spintronic and magnonic applications.

## Graphical abstract

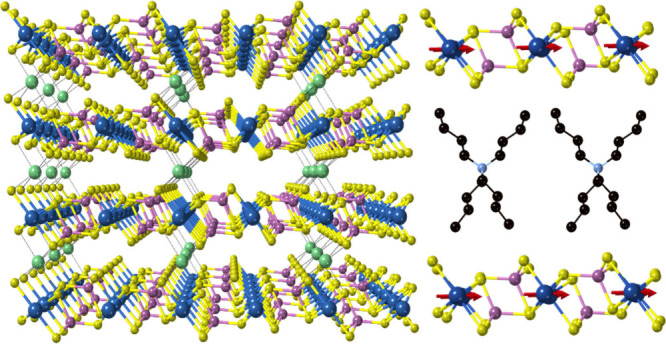

Two-dimensional (2D) van der Waals (vdW) magnets have emerged as ideal platforms to explore low-dimensional magnetism and hold great promise as potential candidates for spintronics, data storage, and sensing applications.^1–4^ Their reduced dimensionality and high surface-to-volume ratio allow fine-tuned control of their properties under external stimuli such as strain, pressure, molecular deposition, electrostatic gating, or twisting.^5–14^ Among the existing 2D vdW magnetic materials, CrSBr and CrPS_4_ stand out due to their and semiconducting character and distinct magnetic anisotropies.^15–23^ CrPS_4_, in particular, is an air-stable layered A-type antiferromagnet that has been successfully integrated into field-effect transistors, allowing gate voltage and perpendicular electric field control of its Néel temperature (*T*_N_), band structure, and magnetization direction.^24–26^ Moreover, long-distance magnon transport and significant modulation of thermal spin currents have been demonstrated in this system.^27–31^ Due to the weak vdW interactions between layers in these compounds, the intercalation of chemical species into the interlayer gaps offers a promising route to manipulate its magnetic behavior.

Among the available tuning strategies, intercalation—the insertion of atoms, ions, or molecules into the vdW gaps—provides a powerful method to modulate interlayer interactions, induce charge transfer and modify the electronic structure.^32–35^ This approach has enabled precise control over superconductivity, electronic transport, and magnetic properties by inducing interlayer expansion, charge transfer between host and guest species, orbital hybridization, or phonon scattering.^36–38^ For instance, Cu intercalation in 2H-NbS_2_ enhances electrical conductivity while suppressing superconductivity,^39^ and K intercalation in 2H-MoS_2_ triggers a structural phase transition to the 1T and 1T′ phases, leading to superconducting behavior.^40^ In vdW magnetic materials, Li^+^ intercalation in CrI_3_ boosts its Curie temperature (*T*_C_),^41^ whereas in CrSBr, it simultaneously increases the electrical conductivity and magnetic ordering temperature and drives an antiferromagnetic (AF)-to-ferromagnetic (FM) phase transition.^42–44^ Beyond alkali metals, organic cations introduce additional tunability due to their structural complexity, enabling both control of the doping level and selective modulation of interlayer spacing.^45,46^ Specifically, intercalation of organic cations in NiPS_3_ decouples the magnetic layers, induces ferrimagnetic order, and shifts its ordering temperature.^47^ Furthermore, the incorporation of tetrabutylammonium (TBA^+^) in Cr_2_Ge_2_Te_6_, Fe_3_GeTe_2_, and CrSBr enhances their ordering temperatures, with Cr_2_Ge_2_Te_6_ also exhibiting a reorientation of its magnetization axis.^43,48,49^

Despite the demonstrated potential of ion intercalation to tune the properties of 2D vdW magnets, its impact on CrPS_4_ remains largely unexplored. In this work, we investigate the effects of Li^+^ and organic TBA^+^ intercalation in CrPS_4_ using first-principles calculations. We show that these guest species modulate the electronic structure and selectively tune intralayer and interlayer exchange interactions, leading to enhanced magnetic ordering temperature, reorientated magnetization easy axis direction and modified magnon transport. Our results establish intercalation as a versatile design strategy for fine-tuning the electronic and magnetic properties in 2D vdW magnets.

CrPS_4_ crystallizes in a monoclinic structure with space group *C*_2_ (No. 5), where Cr and P atoms are coordinated in distorted octahedral and tetrahedral environments, respectively. The octahedral crystal field around the Cr atoms splits the d orbitals into lower-energy t_2g_ (d_*xy*_, d_*xz*_, and d_*yz*_) and higher-energy e_g_ (d_*z* ^2^_ and d_*x* ^2^–*y* ^2^_) states.^20,50^ Magnetically, CrPS_4_ exhibits an A-type AF ground state, composed of FM layers coupled antiferromagnetically along the *c* axis (Figure 1a), with a Néel temperature of *T*_N_ = 38 K.^18^ To gain insight into the electronic and magnetic properties of bulk CrPS_4_, we perform first-principles calculations within the DFT+*U* framework, including a Hubbard correction to account for on-site Coulomb interactions. The optimized lattice parameters (*a* = 10.89 Å, *b* = 7.29 Å, *c* = 6.13 Å, and β = 92°) are in good agreement with prior studies.^51,52^ The computed magnetic moment for each Cr atom is 2.92 μ_B_, consistent with the expected *S* = ^3^/_2_ for Cr^3+^ ions and closely matching the experimentally value of 2.8 μ_B_.^18^ Band structure calculations confirm the semiconducting behavior of CrPS_4_ (Figure 1c), yielding an estimated band gap of 0.87 eV. This value agrees well with scanning tunneling spectroscopy measurements and previous theoretical results.^24,53^ The gap arises from a spin-split band structure, with the valence band maximum and conduction band minimum exhibiting opposite spin character (Figure S1),^50,52–54^ which is confirmed using hybrid functionals (Figure S2). The last occupied valence band shows a maximum at Γ and disperses toward M, as observed in recent angle-resolved photoemission spectroscopy measurements,^55^ while the first unoccupied band shows a large bandwidth of 1 eV (Figure S1), consistent with previous theoretical reports and magnetotransport findings.^24,26^ Orbital-resolved band structure analysis (Figure 1c) indicates that Cr d orbitals dominate near the Fermi level, accompanied by significant contributions from p orbitals of S atoms. We determine the work function (ϕ) of CrPS_4_ and obtain ϕ = 5.3 eV (Figures S3 and S4), aligning well with the experimental reported value of ϕ = 4.9 ± 0.2 eV.^55^

**Figure 1:**
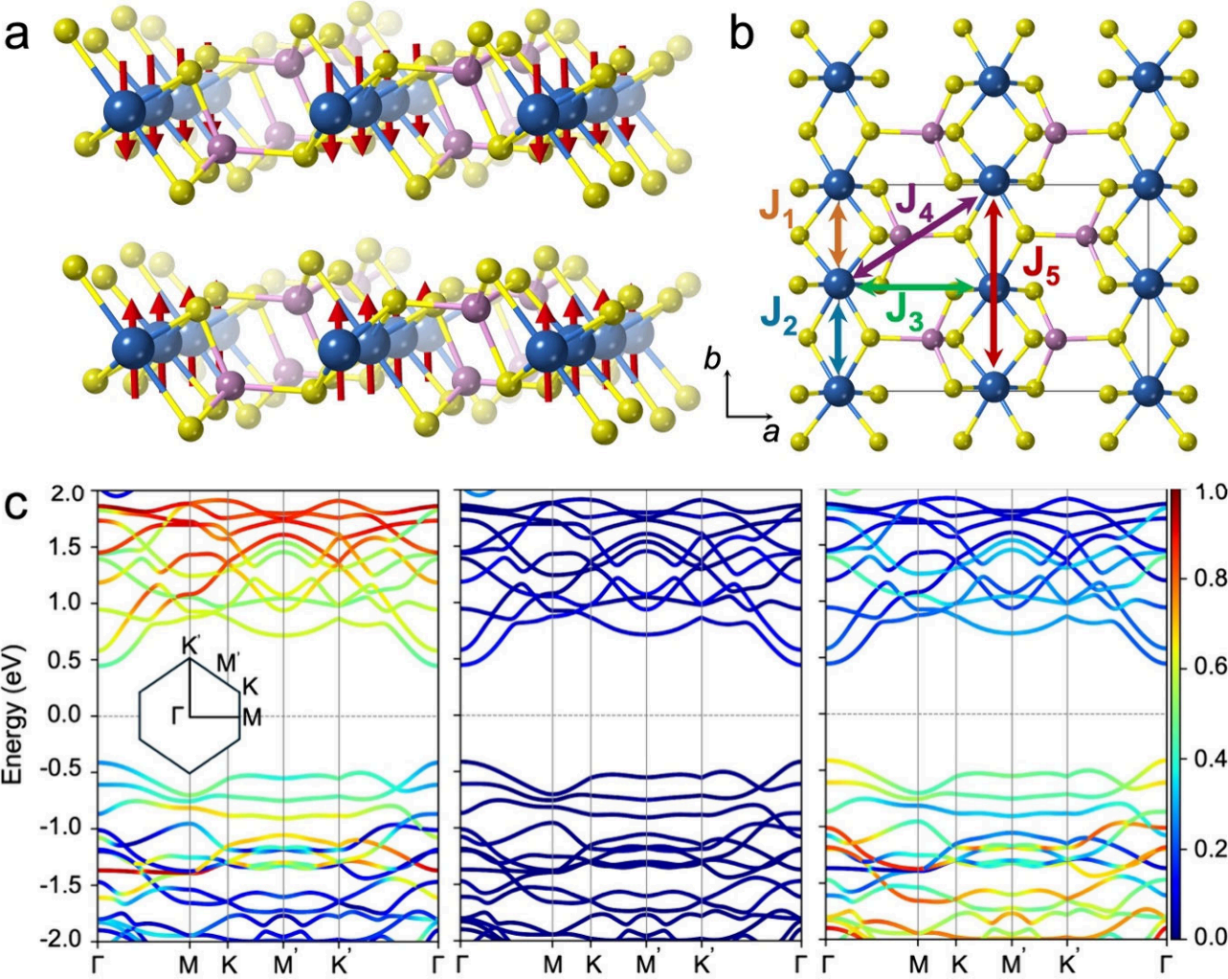
Structural, electronic, and magnetic properties of bulk and single-layer CrPS_4_. (a) Lateral view of bulk CrPS_4_ exhibiting AF ground state, where the red arrows indicate the spin orientation. Color code: Cr, blue; P, pink; S, yellow. (b) Top view of single-layer CrPS_4_ indicating the intralayer magnetic exchange interactions *J*_1_–*J*_5_. (c) Orbital-resolved band structure of bulk CrPS_4_ showing the partial contribution of Cr (d orbitals), P (p orbitals), and S (p orbitals), from left to right, respectively. The color bar indicates the normalized relative contribution to the electronic bands of each orbital.

The magnetic behavior of CrPS_4_ is governed by five dominant intralayer magnetic exchange interactions, *J*_1_–*J*_5_ (Figure 1b). These are extracted by constructing a tight-binding Hamiltonian based on maximally localized Wannier functions (Supporting Information Section 1.2), yielding *J*_1_ = 2.85 meV, *J*_2_ = 2.59 meV, *J*_3_ = 0.05 meV, *J*_4_ = 1.11 meV, and *J*_5_ = −0.83 meV, where positive values denote FM coupling. The larger magnitude of *J*_1_ relative to *J*_2_ arises from the shorter Cr–Cr distance (3.59 vs 3.70 Å) and the Cr–S–Cr bond angle close to 90° (94.98° vs 98.07°), favoring FM superexchange. The biquadratic interactions are also evaluated and found to be negligible compared to bilinear terms (Supporting Information Section 1.3), as observed in the related compound MnPS_3_.^56,57^ On the other hand, interlayer couplings are AF but considerably weaker than the dominant intralayer FM interactions (Figure S5 and Tables S1 and S2). To quantify the effective interlayer exchange (*J*_int_), we calculate the total energy difference between AF and FM spin configurations, defined as *J*_int_ = *E*_AF_ – *E*_FM_. We estimate *J*_int_ = −1.06 meV/Cr, which correctly reproduces the A-type AF ground state of bulk CrPS_4_.^51^

This agrees well with the reported experimental value of −0.58 meV/Cr for bilayer CrPS_4_, where the 2-fold enhancement in the bulk relative to the bilayer is consistent with their different spin-flip fields of 7–8 and 3.5 T, respectively.^18,24,25^

To evaluate the magnetic anisotropy of CrPS_4_, we consider two primary contributions: (i) the spin–orbit coupling term (SOC-MAE) and (ii) the shape anisotropy (shape-MAE), which arises from dipole–dipole interactions. While shape-MAE is typically weaker than SOC-MAE in most 2D magnets, it becomes non-negligible in systems such as CrPS_4_ or CrSBr,^58^ where the orbital moment of Cr^3+^ ions is quenched due to half-filling of the low-energy t_2g_ orbitals. The dipole–dipole contribution is calculated accounting for the discrete spin positions and is particularly relevant at short distances (Supporting Information Section 1.4). This method has shown to capture the magnetic properties of bulk AF and monolayer FM CrSBr^8,17,58,59^ and does not rely on the equilibrium magnetization or the macroscopic sample shape. Considering contributions of both SOC-MAE and shape-MAE, we obtain MAE_*bc*_ = *E*_*b*_ – *E*_*c*_ = 24.5 μeV/Cr and MAE_*ac*_ = *E*_*a*_ – *E*_*c*_ = 39.8 μeV/Cr (Tables S3 and S4). Our results reveal a preferential out-of-plane spin orientations, where *b* and *a* axes are the intermediate and hard magnetization directions, respectively, in agreement with experimental findings.^18^ Specifically, we find that the out-of-plane anisotropy in CrPS_4_ is predominantly stabilized by Cr d orbitals, with an additional but more subtle contribution of p orbitals of S atoms (Figure S8). Atomistic spin simulations based on the extracted exchange couplings and magnetic anisotropy yield a *T*_N_ = 39 K, in excellent agreement with the experimental result of 38 K, thus validating the theoretical framework.^18^ We also examine the evolution of the electronic and magnetic properties of CrPS_4_ as a function of Hubbard *U*, showing that increasing *U* enhances both the electronic band gap and *T*_N_ (Supporting Information Section 1.5).

Then, we investigate the intercalation of Li^+^ ions into the vdW gap of CrPS_4_ (Figure 2a) by considering compositions Li_0.125_CrPS_4_, Li_0.25_CrPS_4_, Li_0.375_CrPS_4_, and Li_0.5_CrPS_4_. Higher concentrations are not studied because it represents the upper limit in related layered materials such as FePS_3_ and NiPS_3_, beyond which structural degradation and eventual breakdown of the samples have been observed.^60,61^ The stability of Li^+^ intercalation is evaluated at multiple sites within the vdW gap (Figures S13 and S14), and in all cases, the intercalation is energetically favorable (Table S6) based on the results of adsorption energy (*E*_ads_). Among them, Li^+^ preferentially occupies positions above the Cr–Cr hollow along the *J*_1_ direction (Figure S13), showing a value of *E*_ads_ = −7.02 eV. Note that the Li–S distance is ∼2.5 Å, significantly shorter than the typical 3–4 Å vdW spacing, pointing to chemical bonding rather than weak vdW interactions. This is further supported by manually increasing the Li–S separation along the *c* direction, resulting in a less stable configuration (Figure S15).

**Figure 2:**
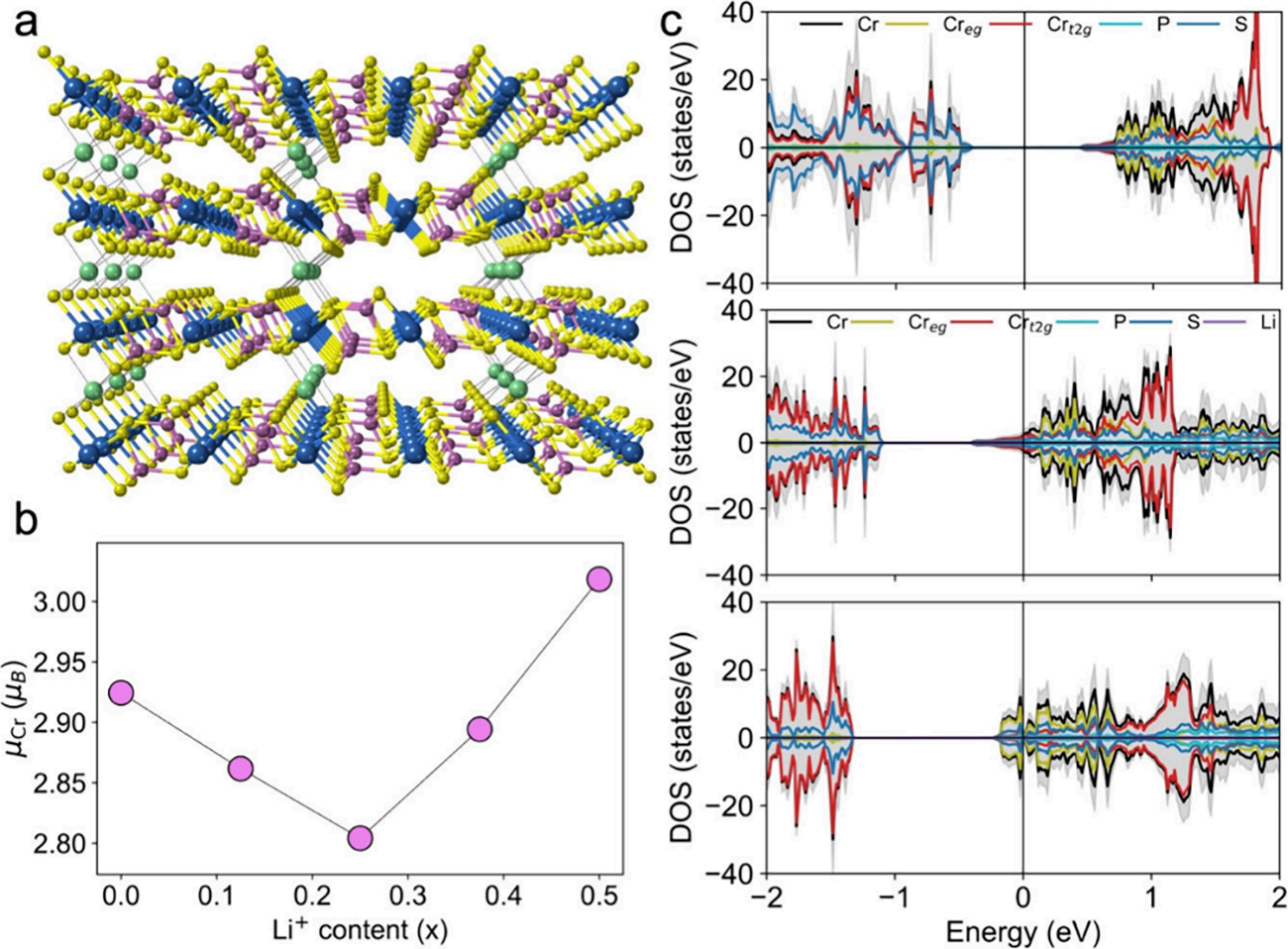
Structural, magnetic, and electronic properties of Li-intercalated CrPS_4_. (a) Lateral view of Li_0.25_CrPS_4_, where green balls depict Li^+^ ions. (b) Evolution of magnetic moments of Cr atoms with increasing Li^+^ content. (c) Orbital-resolved DOS for CrPS_4_, Li_0.25_CrPS_4_, and Li_0.5_CrPS_4_ (from top to bottom, respectively), where the gray filling represents the total DOS.

Upon Li^+^ intercalation, both in-plane lattice parameters as well as the interlayer spacing undergo a progressive expansion (Figure S16), reaching a maximum increase of 1.6%, 1.7% and 1.6% for *a*, *b*, and *c* lattice parameters, respectively, for Li_0.5_CrPS_4_. Conversely, the average Cr magnetic moment exhibits a nonmonotonic evolution, decreasing up to Li_0.25_CrPS_4_ and then increasing for Li_0.375_CrPS_4_ and Li_0.5_CrPS_4_ (Figure 2b). This nonmonotonic trend indicates that, for Li^+^ content ≤ 0.25, the electrons transferred to CrPS_4_ do not reduce Cr^3+^ (d^3^) to Cr^2+^ (d^4^),^62^ which would otherwise enhance the Cr magnetic moments via partial occupation of the empty e_g_ orbitals. This is confirmed by calculating the orbital-resolved density of states (DOS), which shows that in pristine CrPS_4_ the Fermi level resides within the gap, typical of a semiconducting behavior, with unoccupied Cr t_2g_ and S p orbitals located just above the gap (Figure 2c). In Li_0.25_CrPS_4_, the Fermi level shifts into the conduction band, inducing a metallic state. The additional electrons occupy Cr t_2g_ orbitals with opposite spin polarization relative to those at the valence band maximum (Figure S17), leading to a net reduction of the Cr magnetic moment. For Li_0.5_CrPS_4_, spin-up Cr e_g_ states are occupied, resulting in the observed increase in magnetic moments at higher Li^+^ concentrations.

The evolution of interlayer exchange coupling (*J*_int_) reveals a robust AF state up to Li_0.29_CrPS_4_ (Figure 3a), while higher Li^+^ contents (Li_0.29_CrPS_4_–Li_0.5_CrPS_4_) stabilize ferromagnetism through the filling of Cr e_g_ orbitals. Additionally, the out-of-plane magnetic anisotropy softens progressively, driving a reorientation to in-plane magnetization with spins pointing along the *a* axis for Li^+^ contents ≥ 0.17. For higher Li^+^ concentrations (Li_0.4_CrPS_4_–Li_0.5_CrPS_4_), the *b* axis becomes the intermediate magnetization direction, while the *c* axis is the hard magnetic state (Figure S18 and S19). This defines a phase diagram with three different regions: (i) an AF out-of-plane state resembling pristine CrPS_4_ at low Li^+^ content (green), (ii) an AF in-plane state for Li_0.17_CrPS_4_–Li_0.29_CrPS_4_ (blue), and (iii) an ultimate in-plane FM configuration for higher Li^+^ concentrations emerging upon occupation of Cr e_g_ orbitals (red). From the evolution of the intralayer *J*_1_–*J*_5_ exchange couplings one can observe that all are enhanced upon intercalation (Figure 3b). This can be attributed to the additional electrons introduced by Li^+^ ions, leading to a significant strengthening of magnetic couplings.^48^ As a result, the magnetic ordering temperature increases from *T*_N_ = 39K in pristine CrPS_4_ to a maximum of *T*_C_ = 228 K for Li_0.5_CrPS_4_ (Figure 3c).

**Figure 3:**
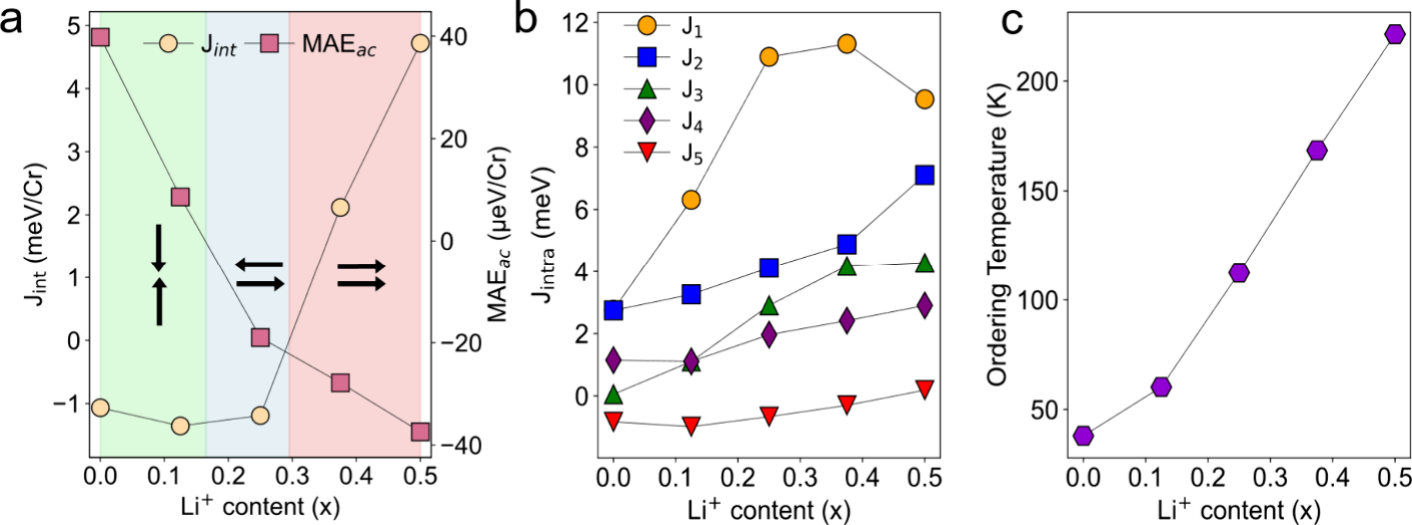
Evolution of the exchange interactions, magnetic anisotropy, and ordering temperature for Li_*x*_CrPS_4_. (a) Evolution of interlayer exchange interaction (*J*_int_) and MAE, where the black arrows indicate the magnetic ground state, (b) intralayer couplings *J*_1_–*J*_5_, and (c) magnetic ordering temperature for Li_*x*_CrPS_4_.

To gain deeper insights into the mechanisms underlying the observed magnetic behavior, we obtain the evolution of the magnetic properties of CrPS_4_ as a function of electron doping, considering purely electrostatic effects and in the absence of intercalated Li^+^ ions. This approach allow us to decouple the effects of (i) Fermi level shifts driven by the additional electrons from (ii) the structural distortions induced by Li^+^ intercalation. Figure S20 shows that electron doping initially reduces the Cr magnetic moments, keeping *J*_int_ nearly constant. Upon occupation of the e_g_ orbitals the magnetic moments increase, rapidly stabilizing interlayer ferromagnetism at 0.42 e^–^/f.u. (Figure S21). Note that the stabilization of the interlayer ferromagnetism occurs independently of the chosen Hubbard *U*, resulting in an earlier FM ground state for larger *U* values (Figure S21). Since each Li^+^ ion ideally donates one electron to CrPS_4_ (e.g., Li_0.5_CrPS_4_ transfers 0.5 e^–^/f.u.), higher electron densities are required to induce interlayer ferromagnetism via electrostatic doping than via Li^+^ intercalation (0.42 vs 0.29 e^–^/f.u., respectively). This difference arises from the expansion of the interlayer spacing upon Li^+^ incorporation, which weakens the AF interlayer coupling and reduces the number of electrons needed to stabilize the FM state. The added carriers gradually reduce the out-of-plane magnetic anisotropy by amplifying the in-plane contributions associated with the (d_*x* ^2^–*y* ^2^_, d_*xy*_) orbitals, ultimately stabilizing in-plane magnetism with enhanced ordering temperature (Figures S22–S26). As electron doping increases and CrPS_4_ becomes metallic, the biquadratic contribution becomes more noticeable; however, it remains small and the bilinear interactions dominate (Figure S27). Due to the similar trends observed for Li^+^ intercalation and electrostatic doping, we infer that carrier injection is the primary mechanism governing the observed magnetic behavior.

Then, we investigate the effect of intercalating a guest molecular cation, specifically the TBA^+^ ion. Two intercalation levels are considered, namely, (TBA)_0.125_CrPS_4_ and (TBA)_0.25_CrPS_4_, corresponding to concentrations that have been experimentally achieved in (TBA)_0.25_NiPS_3_.^47^ The intercalation induces a pronounced expansion of vdW gap, with the interlayer spacing increasing from 2.5 Å in pristine CrPS_4_ to 14.2 Å in the intercalated compounds (Figure 4a). Concurrently, a slight compression of the in-plane lattice parameters is observed, resulting in values of *a* = 10.82 Å and *b* = 7.24 Å for (TBA)_0.125_CrPS_4_ and *a* = 10.75 Å and *b* = 7.20 Å for (TBA)_0.25_CrPS_4_. We perform charge density difference (CDD) calculations to investigate the charge redistribution across the heterostructure (Figure 4a). Our results indicate electron depletion primarily at the terminal hydrogen atoms of TBA^+^ cations, whereas electron accumulation is localized on adjacent S atoms of CrPS_4_, consistent with their higher electronegativity and their closer proximity to TBA^+^. Bader charge-transfer analysis reveals that each TBA^+^ molecule donates 0.90 e^–^ to the substrate (Table S7), inducing a metallic behavior through partial occupation of previously empty conduction bands (Figure S28). Given that in CrPS_4_ the Cr^3+^ ions are coordinated by a distorted S octahedral environment, both species form covalent bonds. As a result, the electrons gained by the S atoms are shared with the Cr atoms, directly influencing their orbital occupations and, consequently, their magnetic moments. Specifically, these are 2.85 μ_B_/Cr and 2.79 μ_B_/Cr for TBA^+^ contents of 0.125 and 0.25, respectively, compared to 2.92 μ_B_/Cr in pristine CrPS_4_. This variation mirrors the prior observations in Li-intercalated CrPS_4_ and is attributed to the occupation of empty t_2g_ states due to charge transfer (Figure S28).

**Figure 4:**
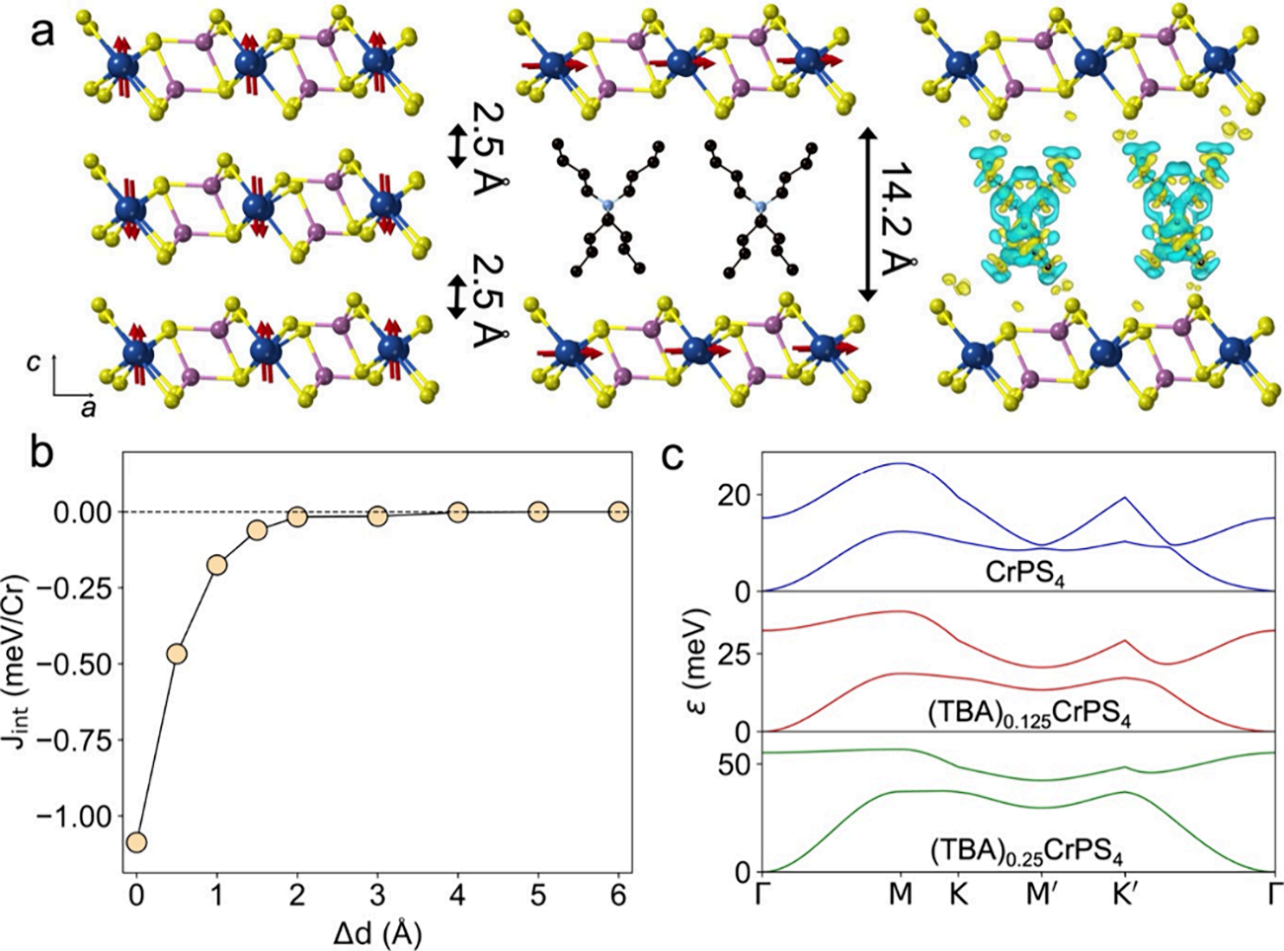
Structural and magnetic properties as well as magnon dispersion of (TBA)_*x*_CrPS_4_. (a) Side views of pristine CrPS_4_ and (TBA)_0.25_CrPS_4_, showing the interlayer spacing and magnetization orientation in each case, together with CDD plots for the latter, where yellow (blue) denotes electron accumulation (depletion). (b) Evolution of *J*_int_ as a function of Δ*d* for pristine CrPS_4_. (c) Magnon dispersion for pristine CrPS_4_, (TBA)_0.125_CrPS_4_, and (TBA)_0.25_CrPS_4_, from top to bottom, respectively.

TBA^+^ incorporation effectively decouples the CrPS_4_ layers along the *c* axis, resulting in magnetically isolated layers, each one exhibiting a FM ground state. To illustrate this, we compute the variation of *J*_int_ for pristine CrPS_4_ as a function of the change in the interlayer distance (Δ*d*). As shown in Figure 4b, the AF coupling rapidly decreases with increasing distance and saturates at Δ*d* ≈ 3 Å. Because the interlayer separation in (TBA)_*x*_CrPS_4_ exceeds this value (Δ*d* = 11.7 Å), *J*_int_ is effectively suppressed. Interestingly, we observe that in the intercalated compounds the magnetization is switched to the in-plane *a* direction, stabilized by the transferred electrons from TBA^+^ to the substrate. Particularly, MAE_*ac*_ changes from 39.8 μeV/Cr (CrPS_4_) to −25.4 and −48.6 μeV/Cr for TBA^+^ contents of 0.125 and 0.25, respectively (Table S8). Upon intercalation, the FM intralayer exchange interactions are enhanced (Table S9), resulting in *T*_C_ of 51 K for (TBA)_0.125_CrPS_4_ and 104 K for (TBA)_0.25_CrPS_4_.

Within 2D magnets, CrPS_4_ has been at the forefront of research on magnon excitations,^27,63,64^ including experimental observations of magnon propagation over large distances of ∼1 μm.^27,64^ The magnon spectrum of CrPS_4_ comprises four magnon branches (Figures 4c and S29),^31^ with the two low-energy bands that would be degenerated within the Heisenberg approximation split due to the triaxial anisotropy of the material (Figures S30 and S31). Anisotropy affects the magnon group velocities, with its most pronounced effect being the suppression of the finite group velocity at Γ due to the induced magnon gap and the quadratic dispersion around this point (Figures S30 and S31).^65^ As shown in Figure 4c, the magnon dispersion predominantly exhibits quadratic behavior along the Γ–M–K–M′–K′−Γ path, characteristic of FM materials. This is consistent with inelastic neutron scattering measurements on similar A-type antiferromagnets such as CrCl_3_ and CrI_3_ ^66,67^ and originates from the dominant in-plane FM intralayer couplings compared to the weaker interlayer AF exchange, in contrast to the linear dispersion observed for conventional three-dimensional antiferromagnets.^68,69^ Notably, at the M′ point and along Γ–K′ direction, the acoustic and optical branches are nearly degenerate due to the near equality of the exchange interactions *J*_1_ (2.85 meV) and *J*_2_ (2.59 meV). Furthermore, the energy of acoustic magnons increase more rapidly along the y direction (Γ–K′) than along *x* (Γ–M), resulting in maximum group velocities of *v*_*y*_ = 8.4 × 10^3^ m·s^–1^ and *v*_*x*_ = 4.9 × 10^3^ m·s^–1^, respectively, in agreement with previous findings.^31^ This anisotropy arises from the larger values of *J*_1_ and *J*_2_, which are aligned along *y*, compared to *J*_3_ (0.05 meV) along *x*, while *J*_4_ contributes to both because it points along the diagonal direction (Figure 1). Intercalation with TBA^+^ molecules lifts the degeneracy at M′ and Γ–K′ due to the marked differences between *J*_1_ and *J*_2_ (Table S9) and results in enhanced magnon velocities. For (TBA)_0.125_CrPS_4_, *v*_*y*_ and *v*_*x*_ reach 9.9 × 10^3^ and 7.7 × 10^3^ m·s^–1^, respectively, further increasing to 13.9 × 10^3^ and 15.4 × 10^3^ m·s^–1^ for (TBA)_0.25_CrPS_4_ (Figure S30). This indicates that TBA^+^ cations not only increase magnon velocities but also make their propagation more isotropic, primarily due to the enhanced magnitude of *J*_3_ (Table S9).

Our results highlight molecular intercalation in 2D vdW magnetic materials as a powerful and versatile tool to chemically control magnonic devices. This combines the accessibility of experimental techniques to measure magnons in bulk systems (e.g., magnon dispersion via inelastic neutron scattering)^70,71^ with the interfacial tunability of 2D-layered materials,^72^ enabling precise layer-by-layer modulation of the magnetic properties of the system.

In summary, we have investigated the tunability of the electronic and magnetic properties of CrPS_4_ via ion intercalation using first-principles calculations. We demonstrate that Li^+^ intercalation induces a semiconductor-to-metal transition while selectively modifying magnetic exchange interactions, giving rise to a sequence of magnetic phases: an out-of-plane AF state at low Li^+^ content, an in-plane AF regime at intermediate concentrations, and a FM in-plane ground state at higher intercalation levels. This is rationalized from a microscopic analysis due to the different occupations of CrPS_4_ orbitals upon electron doping. Additionally, Li^+^ intercalation leads to a significant enhancement of the magnetic ordering temperature, reaching *T*_C_ = 228 K for Li_0.5_CrPS_4_, driven by the strengthening of intralayer exchange interactions. On the other hand, the intercalation of organic TBA^+^ cations expand the vdW gap, effectively decoupling CrPS_4_ layers. Notably, the transferred electrons from TBA^+^ modify the electronic structure, inducing metallicity. Furthermore, TBA^+^ intercalation stabilizes in-plane ferromagnetism, resulting in an enhanced *T*_C_ above 100 K, increased magnon group velocities, and a more isotropic magnon transport. Our work provides a microscopic understanding of how chemical species inserted into the interlayer gaps influence electronic and magnetic properties, offering a pathway to design highly tunable 2D magnetic materials for spintronics and magnonic applications.

## Methods

Spin-polarized density functional theory (DFT) calculations for CrPS_4_ were performed using the *VASP* package.^73^ The exchange-correlation energy was treated within the generalized gradient approximation. To accurately describe the electronic and magnetic properties of CrPS_4_, a Hubbard-corrected DFT+*U* approach was employed with a Hubbard *U* value of 0.25 eV. This value of *U* was chosen to best reproduce the experimental electronic band gap and *T*_N_ (see the Supporting Information) and correctly captures the electronic properties obtained using the HSE06 hybrid functional. We employed the DFT-D2 to describe the vdW interactions between adjacent layers of CrPS_4_. Maximally localized Wannier functions were constructed using *Wannier90*,^74^ with the d orbitals of Cr and the p and s orbitals of P and S as the basis, to generate a tight-binding Hamiltonian. Magnetic exchange couplings were subsequently calculated using TB2J code, employing supercells of dimensions 10 × 10 × 5.^75^ *T*_N_ and *T*_C_ were determined through atomistic simulations using the VAMPIRE code.^76^ Simulations were performed on supercells of dimensions 25 nm × 25 nm × 25 nm, with both equilibration and averaging phases performed over 10,000 steps employing the llg-heun integrator.

## Supplementary Material



## Notes

Electronic and magnetic properties of CrPS_4_, Li^+^-intercalated CrPS_4_, and TBA^+^-intercalated CrPS_4_ (PDF)
