# Supplementary material for: Controlling Magnetism in the 2D van der Waals Antiferromagnet CrPS4 via Ion Intercalation
Source: Nano Lett. 2026 Feb 10;26(9):3018–25. doi: 10.1021/acs.nanolett.5c05445 (PMC12983355; doi:10.1021/acs.nanolett.5c05445)
Supplement: Supplementary file 1 [file nl5c05445_si_001.pdf]

Supporting Information

Controlling Magnetism in the 2D van der Waals Antiferromagnet $CrPS_4$ via Ion Intercalation

Authors: Alberto M. Ruiz[†], Diego López-Alcalá[†], Gonzalo Rivero-Carracedo[†], Andrei Shumilin[†], José J. Baldoví[†,*]

[†]Instituto de Ciencia Molecular, Universitat de València, Catedrático José Beltrán 2, 46980 Paterna, Spain. E-mail: j.jaime.baldovi@uv.es

Table of Contents

1. Electronic and magnetic properties of $CrPS_4$ 2
- 1.1 Band structure and work function 2
- 1.2 Comparison of intralayer and interlayer exchange interactions 4
- 1.3 Biquadratic interactions 5
- 1.4 Magnetic anisotropy energy 6
- 1.5 Electronic and magnetic properties vs Hubbard U 8

2. Li^+ intercalated $CrPS_4$ 11
- 2.1 Electron doping effect on $CrPS_4$ 16

3. TBA^+ intercalated $CrPS_4$ 20

1. Electronic and magnetic properties of $CrPS_4$

1.1 Band structure and work function

From the calculation of the band structure, we obtained that the last occupied bands of $CrPS_4$ and the first unoccupied bands show different spins. Note that in the AF configuration such differences cannot be observed, as spin up and down are degenerate. For such purpose, we computed the FM configuration, showing that the last valence bands show spin up component whereas the first occupied states show spin down, feature also captured employing using the HSE06 hybrid functional.[1]

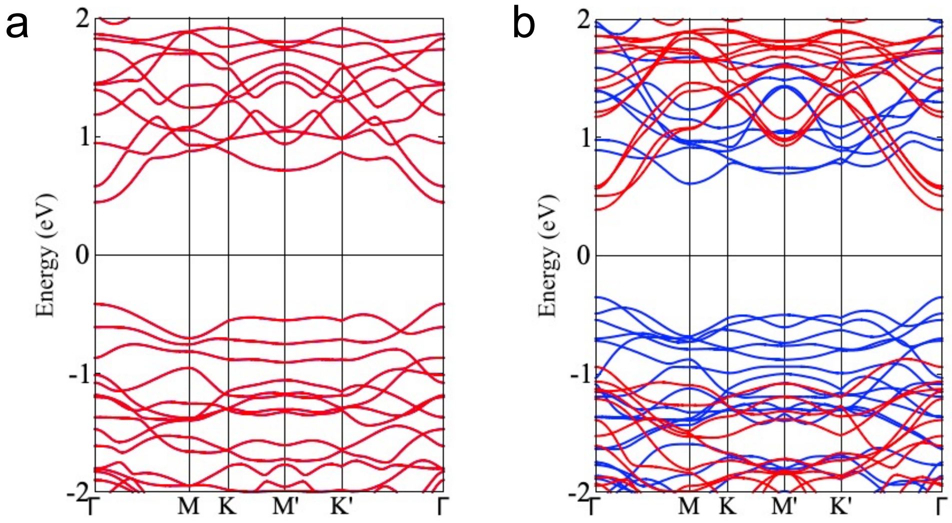

Figure S1. Band structure of bulk $CrPS_4$ in the AF (a) and FM (b) configuration for U = 0.25 eV. Blue and red lines represent spin up and down components, respectively.

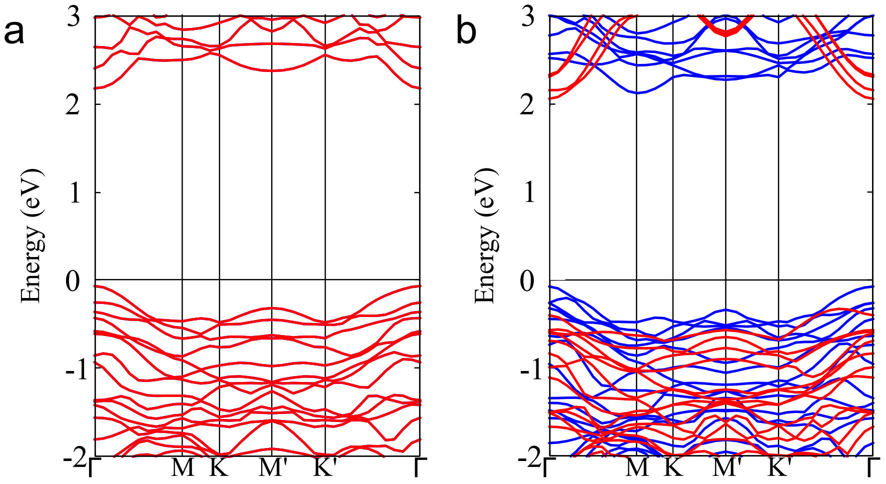

Figure S2. Band structure of bulk $CrPS_4$ in the a) AF and b) FM configuration using the HSE06 hybrid functional. Blue (red) color in the band structure indicates spin up (down) states.

The work function (ϕ) for bulk $CrPS_4$ is determined by considering a slab model of 4 layers. The ϕ is calculated as the difference between the electrostatic potential in the vacuum region, namely vacuum energy (E_{vacuum}), and the Fermi energy.

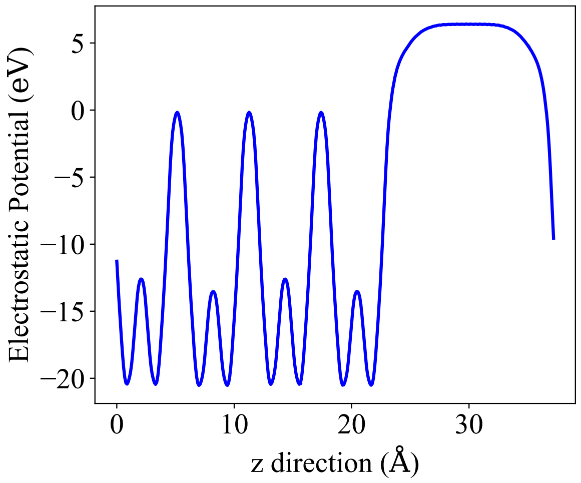

Figure S3. Evolution of the electrostatic potential as a function of the distance along the z direction. The E_{vacuum} = 6.41 eV is determined as the energy from the region at which the electrostatic potential does not change.

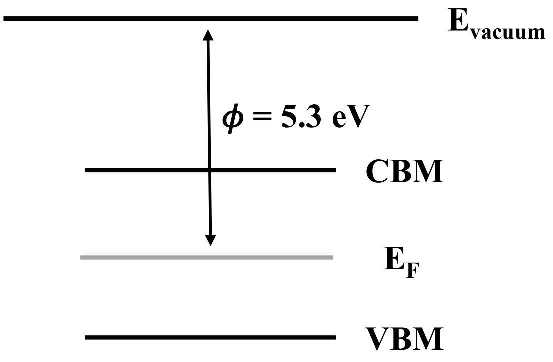

Figure S4. Schematic representation of the extracted work function ϕ calculated as the energy difference between E_{vacuum} and E_F.

1.2 Comparison of intralayer and interlayer exchange interactions

The magnetic exchange interactions were extracted using a spin Hamiltonian with the following form:

$$H = -\sum_{i \neq j} J_{ij} \vec{S}_i \cdot \vec{S}_j \qquad (1)$$

where J_{ij} denotes the isotropic exchange interaction between magnetic moments S_i and S_j.

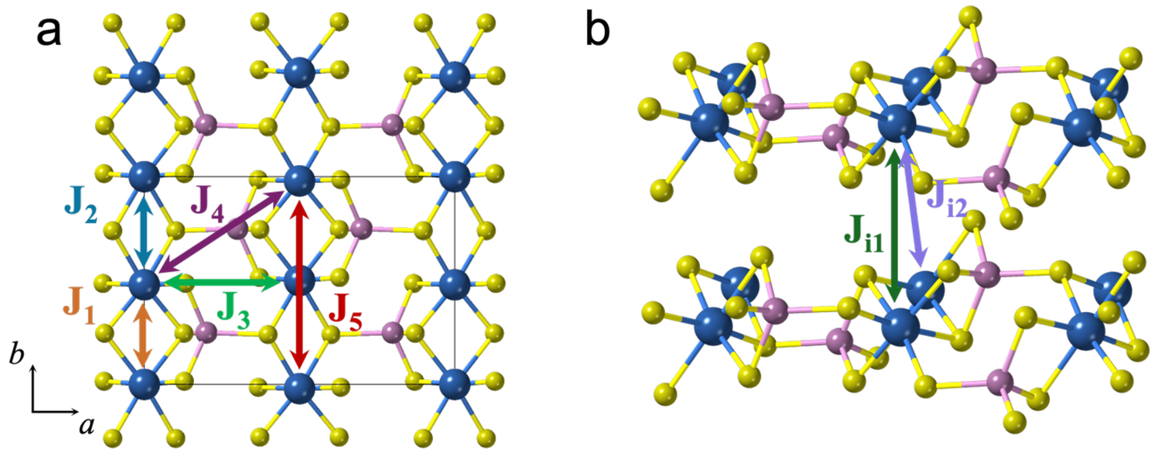

Figure S5. Top a) and lateral b) views of bulk $CrPS_4$ with their corresponding intralayer (J_1-J_5) and interlayer (J_{i1} and J_{i2}) exchange interactions. Color code: Blue, yellow and pink balls represent Cr, S and P atoms, respectively.

Table S1. Values of intralayer exchange interactions J_1-J_5 for $CrPS_4$, along with the number of nearest neighbours (NN) and the distances between them (d).

Interaction	Value (meV)	NN	d (Å)
J_1	2.85	1	3.59
J_2	2.59	1	3.70
J_3	0.05	2	5.45
J_4	1.11	4	6.55
J_5	-0.83	2	7.29

Table S2. Interlayer exchange interactions J_{i1} and J_{i2} for $CrPS_4$ along with the number of nearest neighbours (NN) and the distance between them (d).

Interaction	Value (meV)	NN	d (Å)
J_{i1}	-0.05	2	6.13
J_{i2}	-0.07	2	7.16

1.3 Biquadratic interactions

The biquadratic interactions are evaluated following the procedure described by Kartsev et al.[2] Specifically, we have considered bulk $CrPS_4$ and rotated adjacent spins by θ along the *xz* plane between two known spin configurations: from ferromagnetic (FM) at θ° = 0, to antiferromagnetic (AF) at θ = 180° in steps of 20°.

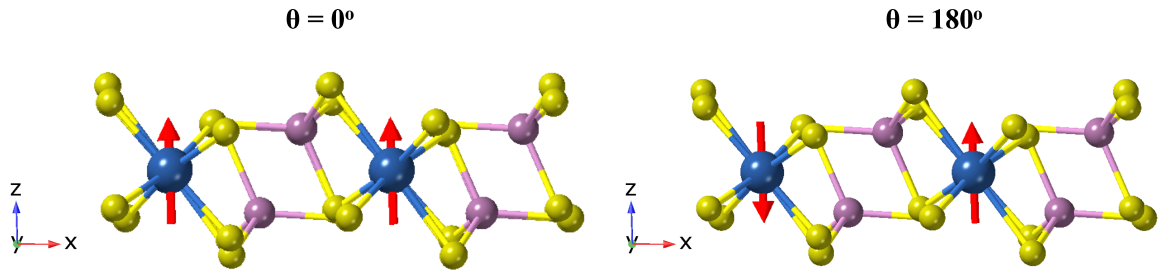

Figure S6. Lateral view of single-layer $CrPS_4$ where the red arrows represent the orientation of magnetic moments of Cr atoms for θ = 0^0 (FM) and 180° (AF).

The results are presented in Figure S7, where one can observe that the total energy increases as one moves from the FM configuration (θ = 0°) to the AF state (θ = 180°), as expected given that $CrPS_4$ shows intralayer FM ground state. Then, we fitted our total energy calculations to a quadratic fitting using:

$$E_{bq}^{tot}(\theta) = A_0^{bq} + A_1^{bq}.S^2 cos(\theta) + A_2^{bq}.S^4 cos^2(\theta) \quad (2)$$

as well as to a linear fitting, the latter using:

$$E_{bl}^{tot}(\theta) = A_0^{bl} + A_1^{bl}.S^2 cos(\theta) \quad (3)$$

We observe that the linear and the biquadratic fitting almost overlap, implying that the material develops small biquadratic exchange interactions compared to bilinear interactions.

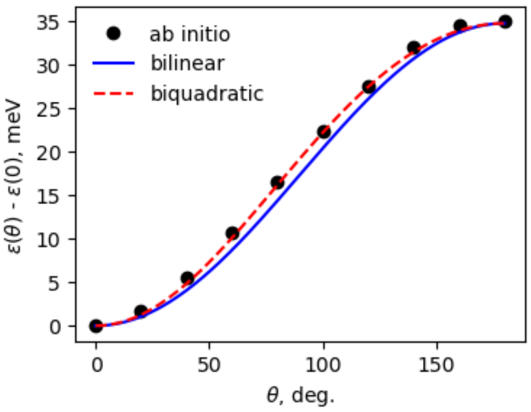

Figure S7. Total energy evolution as a function of rotation angle (θ) for $CrPS_4$ along with the corresponding quadratic (red dashed line) and linear (blue solid line) fittings.

Note that Kartsev et al.[2] report similar results for $MnPS_3$, a compound that shares similarities with $CrPS_4$. Consistent with our findings, $MnPS_3$ exhibits negligible biquadratic interactions, and its behaviour is predominantly governed by bilinear exchange. Amirabbasi et al.[3] drew the same conclusion, showing that $MnPS_3$ displays only a small biquadratic contribution compared to bilinear term and that the biquadratic coupling increases along the transition-metal series (i.e., it is enhanced when moving from $MnPS_3$ to $FePS_3$, $CoPS_3$, and $NiPS_3$).

1.4 Magnetic anisotropy energy

The magnetic anisotropy of $CrPS_4$ is evaluated by considering two primary contributions: (i) the spin–orbit coupling term (SOC-MAE) and (ii) the shape anisotropy (shape-MAE), the latter arising from long-range dipole–dipole interactions. The SOC-MAE is obtained from the total energy difference between in-plane and out-of-plane spin orientations. On the other hand, the shape-MAE is calculated using the following expression:

$$E = \frac{\mu_0}{8\pi}\sum_{i\neq j}\frac{1}{|\overrightarrow{r_{ij}}|^3}\left(\overrightarrow{m_i}.\overrightarrow{m_j} - \frac{3(\overrightarrow{m_i}.\overrightarrow{r_{ij}})(\overrightarrow{m_j}.\overrightarrow{r_{ij}})}{|\overrightarrow{r_{ij}}|^2}\right) \quad (4)$$

where r_{ij} is a vector between two magnetic atoms and m_i and m_j are the magnetic moments of Cr atoms. We calculate the dipole-dipole energy of bulk $CrPS_4$ considering dimensions of N×N×N, where N is the number of unit cells along each crystallographic direction (a, b, c). Therefore, dipole MAE is calculated by the difference of dipole-dipole energy between two directions, resulting in $MAE_{ac} = E_a - E_c$ and $MAE_{bc} = E_c - E_b$, reaching a saturation value with increasing N. We determine that for N = 5 the dipole MAE is already converged with respect to N = 7 (Table S3).

Table S3. Calculated Shape-MAE for bulk $CrPS_4$ using different N×N×N dimensions.

Unit cells	MAE_{bc} (μeV/Cr)	MAE_{ac} (μeV/Cr)
5×5×5	-18.1	-2.5
7×7×7	-18.6	-2.6

In Table S4 we show that the SOC-MAE stabilises the out-of-plane direction, while the dipolar anisotropy favours in-plane alignment. Considering both contributions, we obtain that MAE_{bc} = 24.5 μeV/Cr and MAE_{ac} = 39.8 μeV/Cr. Therefore, our results reveal a preferential out-of-plane spin orientation, where *b* and *a* axes are the intermediate and hard magnetization directions, respectively, in agreement with experimental findings.[4]

Table S4. Values of SOC, Shape and total MAE for $CrPS_4$ expressed with respect to the easy *c* magnetization axis.

Contribution to MAE	MAE_{bc} (μeV/Cr)	MAE_{ac} (μeV/Cr)
SOC	42.6	42.3
Shape	-18.1	-2.5
Total	24.5	39.8

Figure S8 shows that the out-of-plane anisotropy in $CrPS_4$ is predominantly stabilized by Cr-d orbitals, with an additional but more subtle effect of p orbitals of S atoms. Regarding the Cr-d contribution, our results indicate that the out-of-plane anisotropy is mainly stabilized through the coupling between the (d_{yz}, d_{z2}) orbitals, while its magnitude is partially reduced by competing in-plane contributions arising from the (d_{x2-y2}, d_{xy}) orbitals.

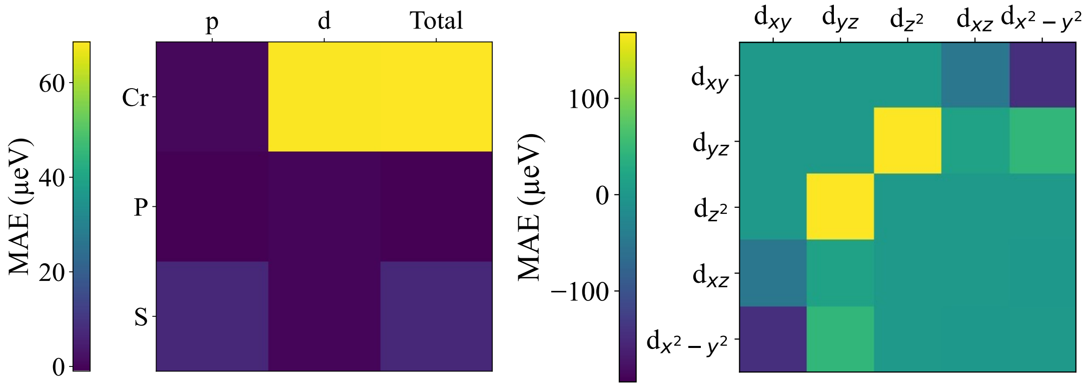

Figure S8. Atomic-resolved (left) and Cr-d orbital-resolved (right) contributions to MAE in $CrPS_4$. Positive and negative values indicate stabilization of out- and in-plane MAE, respectively.

1.5 Electronic and magnetic properties vs Hubbard U

To assess for the impact of Hubbard U in the electronic and magnetic properties of $CrPS_4$, we performed a screening of these properties varying U from 0 to 3 eV. In Figures S9 and S10, we observe that upon increasing U, there is a continuous enhancement of the electronic band gap in both AF and FM configurations. Since this gap has been determined to be 0.87 eV by STS measurements,[5] we find a better agreement at low values of U (0-1 eV), particularly at U = 0.25 eV (Table S5). Additionally, the electronic gap corresponds to the energy difference between the last occupied valence band with spin up component and the lowest unoccupied conduction band with opposite spin, feature that is well captured at 0 eV < U < 1.5 eV (Figure S10). To further corroborate this, we calculate the band structure using the HSE06 hybrid functional in both AF and FM configurations (Figure S11), which show that last occupied valence band and the lowest unoccupied conduction band show different spin behaviour, feature that is properly captured in the low U range 0 eV ≤ U < 1.5 eV.

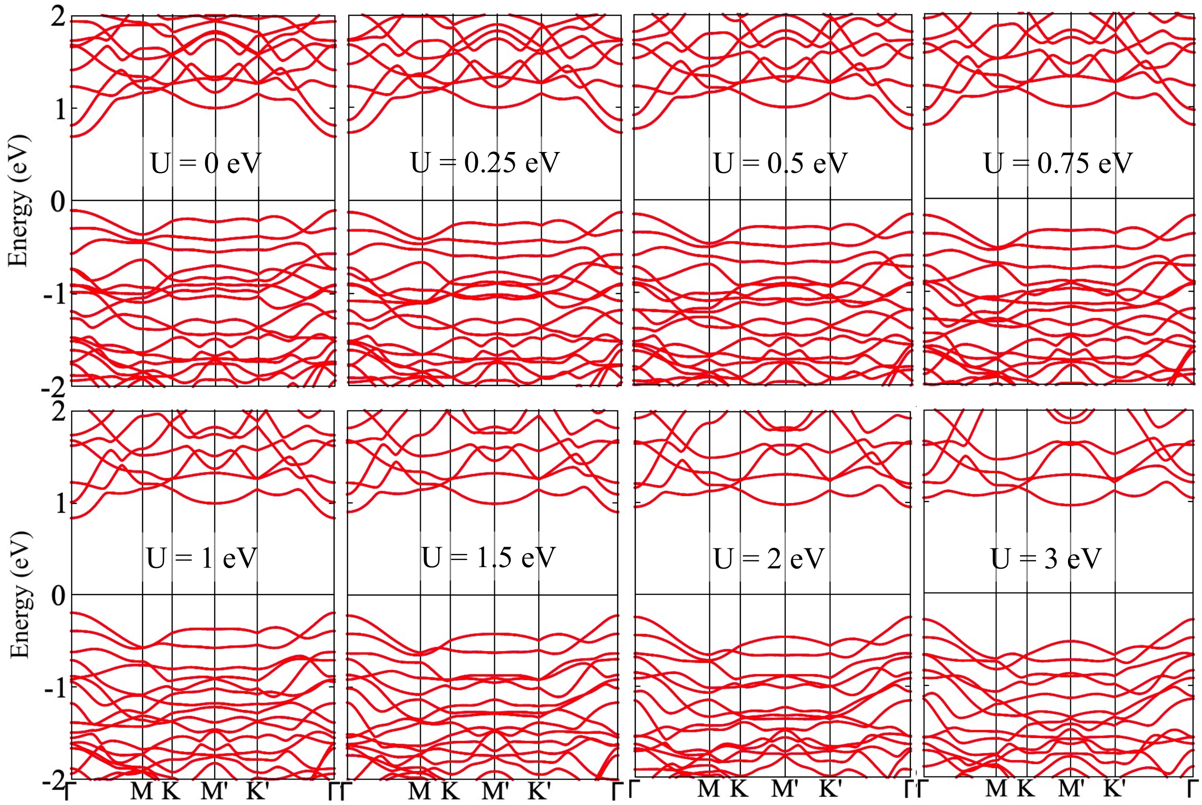

Figure S9. Evolution of band structure of bulk $CrPS_4$ in the AF configuration as a function of Hubbard U. Blue and red lines represent spin up and spin down states.

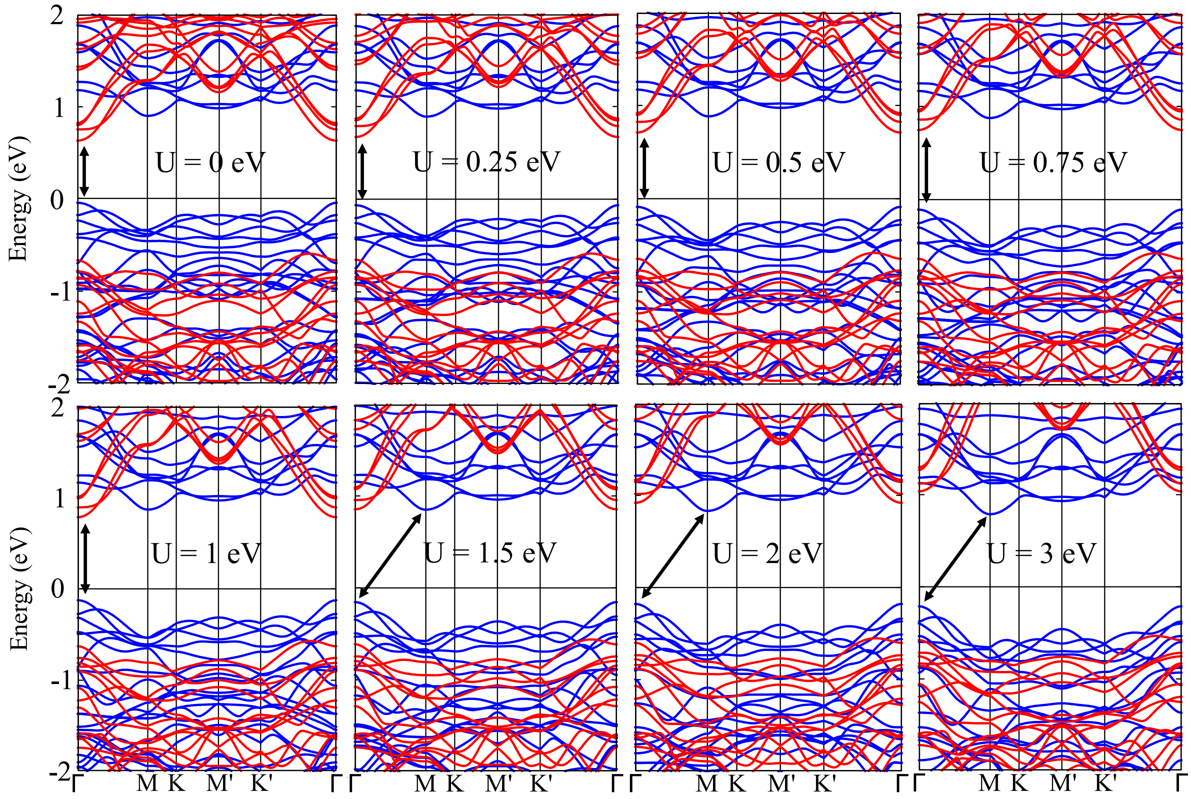

Figure S10. Evolution of band structure of bulk $CrPS_4$ in the FM configuration as a function of Hubbard U. Blue (red) color in the band structure indicates spin up (down) states. Black arrows represent the transition from the last occupied band to the first unoccupied band.

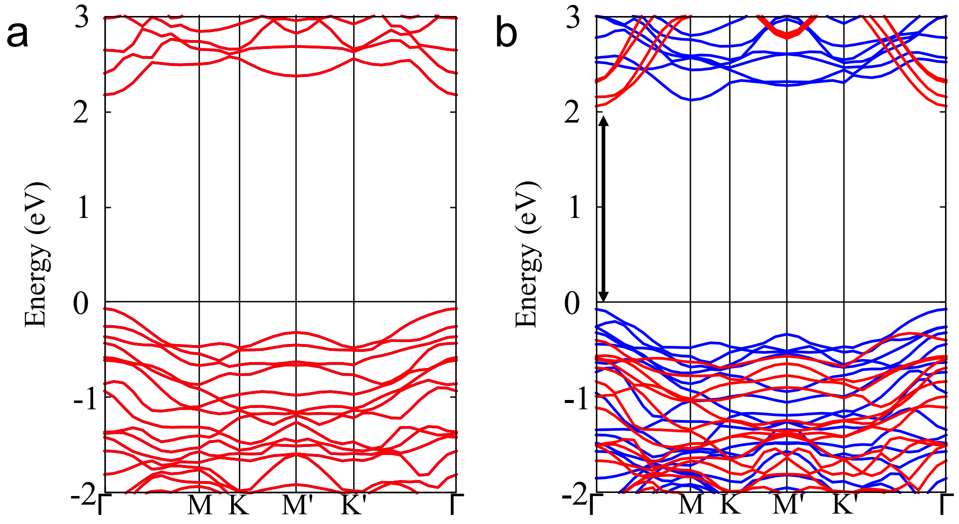

Figure S11. Band structure of bulk $CrPS_4$ in the a) AF and b) FM configuration using the HSE06 hybrid functional. Blue (red) color in the band structure indicates spin up (down) states. Black arrow represents the transition from the last occupied band to the first unoccupied band.

Table S5. Evolution of the electronic band gap for $CrPS_4$ in the AF and FM configurations as a function of Hubbard U and for the hybrid HSE06 functional.

Hubbard U (eV)	Band gap AF (eV)	Band gap FM (eV)
0	0.79	0.68
0.25	0.86	0.75
0.5	0.93	0.82
1	1.02	0.91
1.5	1.12	1.01
2	1.19	1.09
3	1.26	1.14
HSE06 functional	2.24	2.13

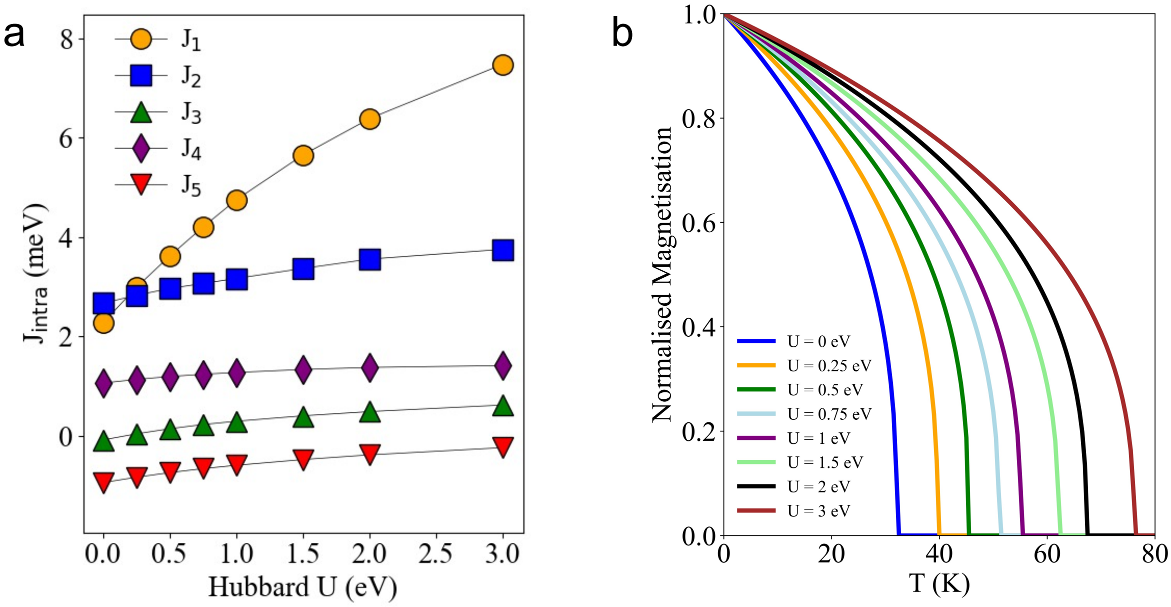

Figure S12. Evolution of a) intralayer exchange couplings J_1-J_5 and b) T_N for $CrPS_4$ as a function of Hubbard U.

Additionally, we computed the evolution of the intralayer exchange couplings J_1-J_5 as a function of U (Figure S12), where we find that all couplings exhibit a continuous enhancement upon increasing U. The most pronounced effect is exhibited by J_1, which increases from 2.27 meV at U = 0 eV to 7.48 meV at U = 3 eV. Consequently, the Néel temperature (T_N) also increases monotonically, from 32 K at U = 0 eV to 76 K at U = 3 eV. Considering the experimental value of T_N = 38 K,[4] the best agreement is obtained at U = 0.25 eV, for which our calculations yield T_N = 39 K. Therefore, we adopt a Hubbard U = 0.25 eV in the subsequent calculations since it correctly captures both electronic and magnetic properties of $CrPS_4$. Similarly, comparable low values of U have been employed for CrSBr, successfully reproducing its AF interlayer coupling and the experimentally observed direct band gap.[6–9]

2. Li^+ intercalated $CrPS_4$

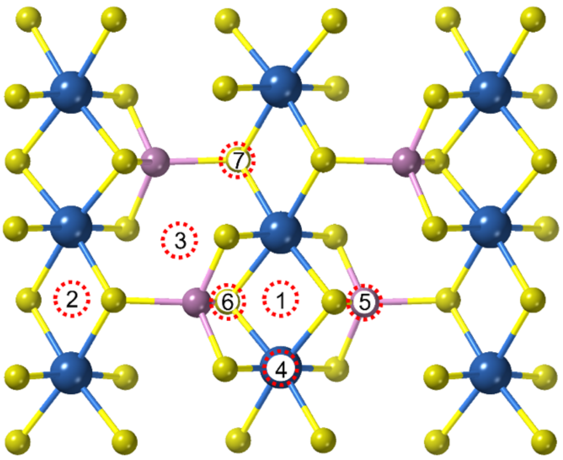

Figure S13. Top view of single-layer $CrPS_4$ numbering the different positions above which Li^+ can be placed.

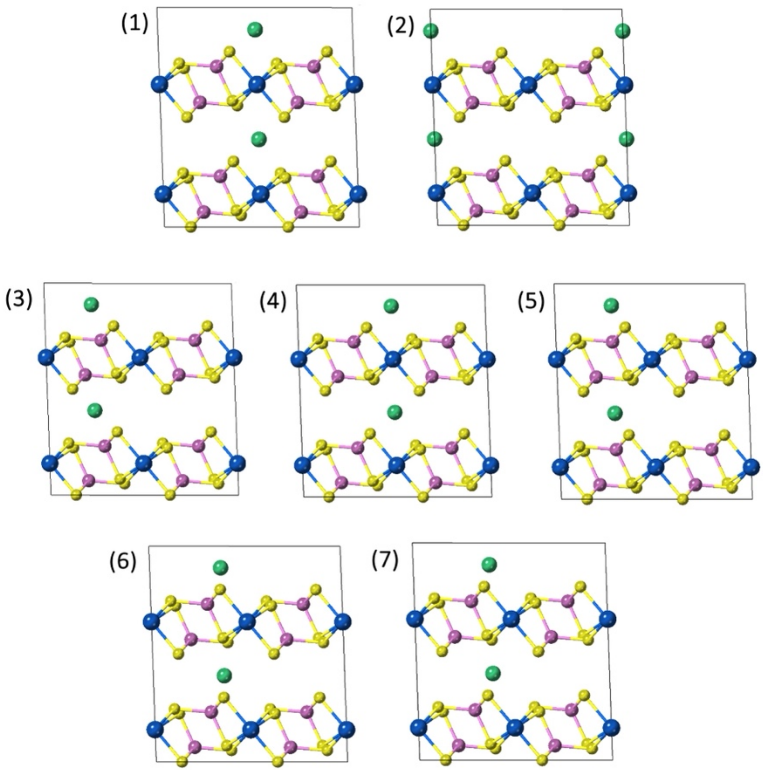

Figure S14. Lateral views of the different configurations for Li_xCrPS_4.

Table S6. Adsorption energy (E_{ads}) and distance to the nearest S atom for the different adsorption configurations for $Li_{0.25}CrPS_4$.

Site	E_{ads} (eV)	Distance to nearest S (Å)
1	-7.02	2.55
2	-5.84	2.38
3	-6.49	2.42
4	-5.89	2.33
5	-6.49	2.42
6	-7.02	2.55
7	-5.84	2.38

In Table S6 we can observe that E_{ads} values are highly negative, which indicates that the intercalation is favoured for every adsorption site and that the resulting system is energetically stable, forming a chemical bond between Li-S. As a general trend, the Li^+ ions tend to maximize the interaction with the S atoms from the substrate. The strongest interactions occur at sites 1 and 6, (E_{ads} = -7.02 eV). In these configurations, the Li^+ ions adopt a position equidistant from four S atoms (2.55 Å). In the remaining configurations, the Li^+ interacts with only two S atoms at comparable distances, leading to slightly less negative E_{ads}. Furthermore, in these less stable configurations, Li^+ is positioned closer to the S atoms (≈2.4 Å), which correlates with a decrease in stability due to a higher repulsion.

Interestingly, although the initial atomic arrangement of site 6 differs from site 1, structural relaxation leads to convergence toward the same optimized configuration observed for site 1. A similar behavior is found for site 7, which relaxes into the geometry of 2, consistent with the comparable E_{ads} values and Li–S bond lengths. Therefore, site 1 represents the most stable configuration, characterized by a Li–S distance of approximately 2.5 Å—significantly shorter than the typical 3–4 Å van der Waals separation—suggesting the formation of chemical bonds rather than weak vdW interactions. This interpretation is further supported by manually increasing the Li–S separation along the *c* direction, resulting in a less stable configuration (Figure S15).

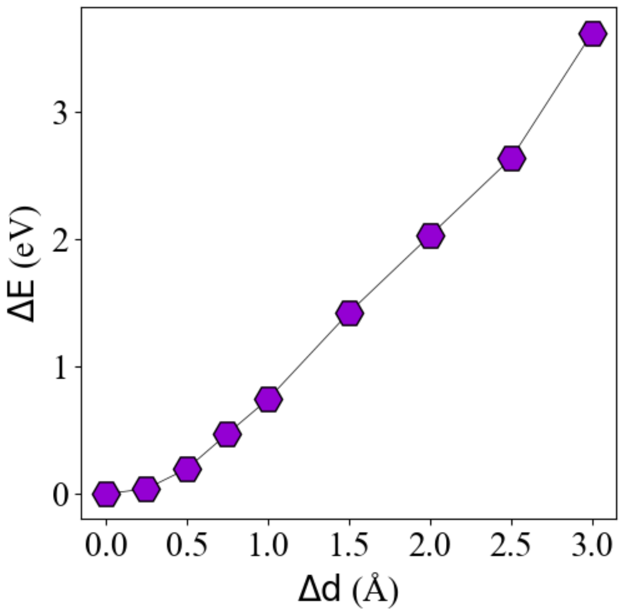

Figure S15. Total energy difference (ΔE) for $Li_{0.25}CrPS_4$ as a function of Li–S separation along the *c* direction relative to the ground state. The results indicate that as the interaction shifts from a chemical Li–S bond to vdW–like interactions, the system drives to a less stable configuration.

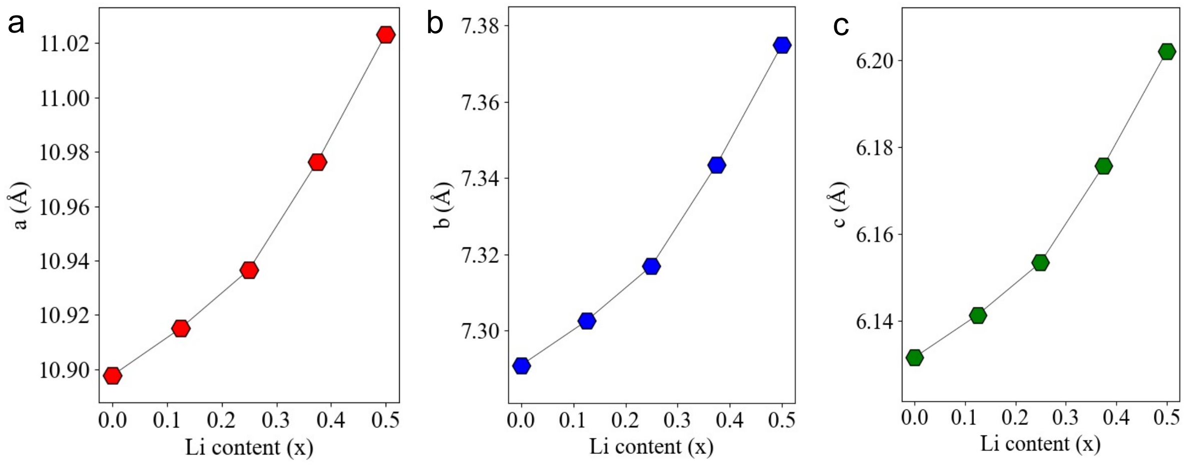

Figure S16. Evolution of a) *a*, b) *b* and c) *c* lattice parameters as a function of Li^+ content.

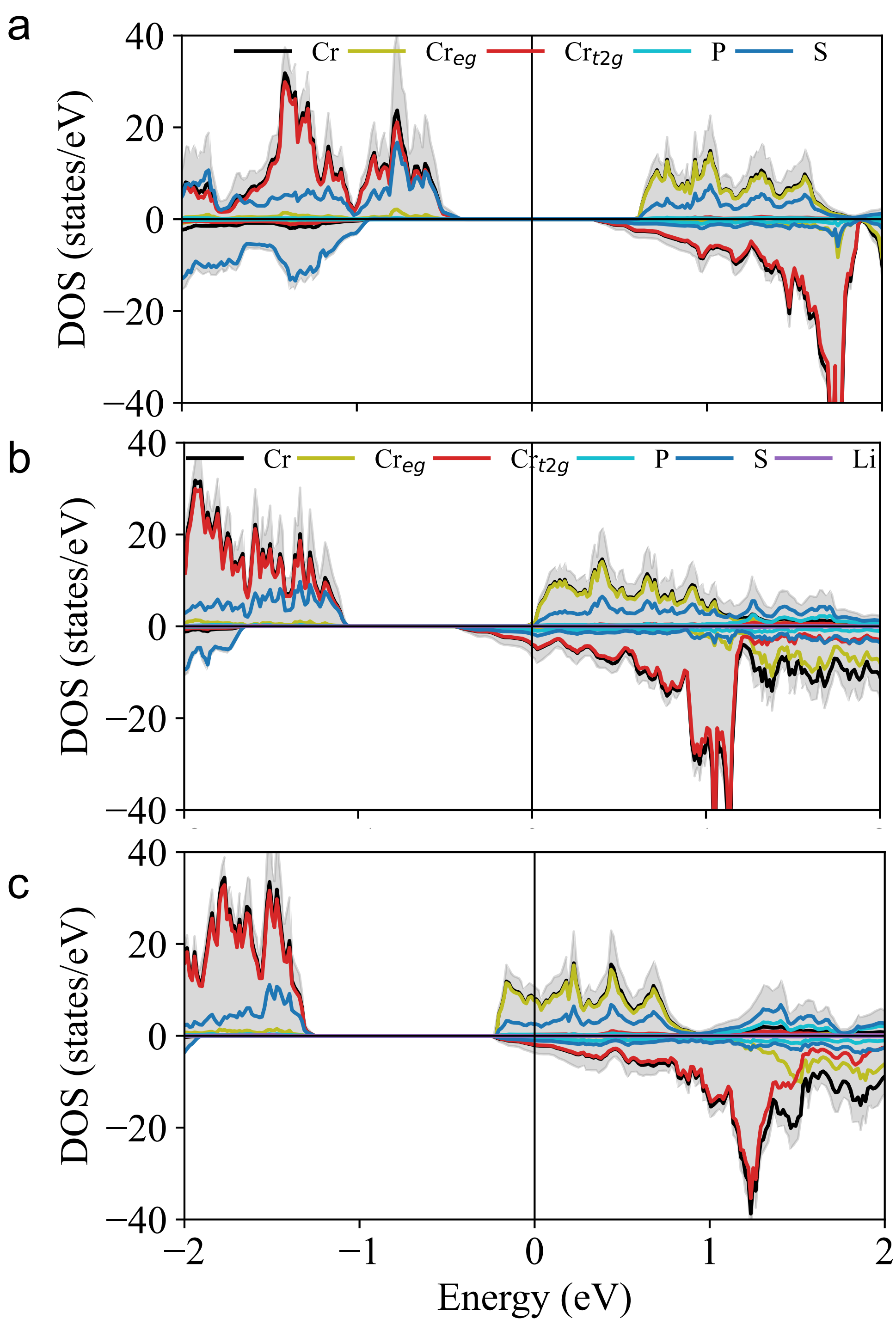

Figure S17. Orbital resolved DOS for (a) $CrPS_4$, (b) $Li_{0.25}CrPS_4$ and (c) $Li_{0.5}CrPS_4$ in the FM configuration.

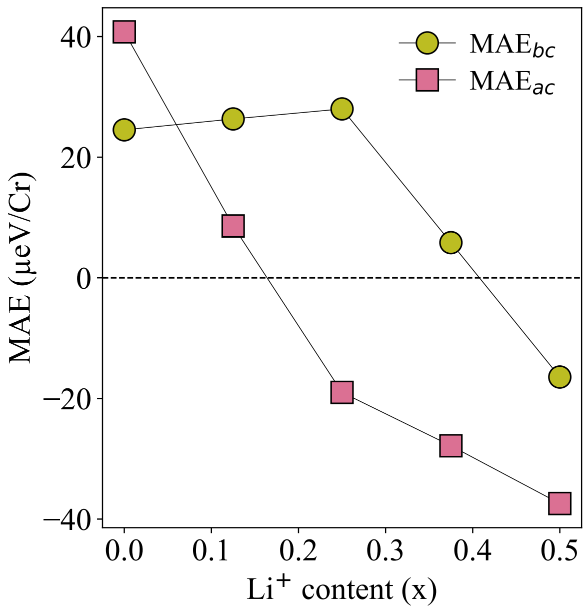

Figure S18. Evolution of MAE_{bc} and MAE_{ac} as a function of Li^+ content.

Figure S18 illustrates that pristine $CrPS_4$ exhibits a ground state with spins oriented along the *c* axis, while the *b* and *a* axes correspond to the intermediate and hard magnetization directions, respectively. Upon incorporation of Li^+ ions, the *a* axis becomes progressively stabilized, leading to a spin reorientation toward magnetization along the *a* axis for Li^+ contents ≥ 0.17. In the range $0.17 \leq x \leq 0.4$, the spins remain aligned along the *a* axis, while *c* and *b* axes correspond to the intermediate and hard magnetization directions, respectively. For Li^+ contents exceeding 0.4, *b* is favoured over the *c* axis as the intermediate direction.

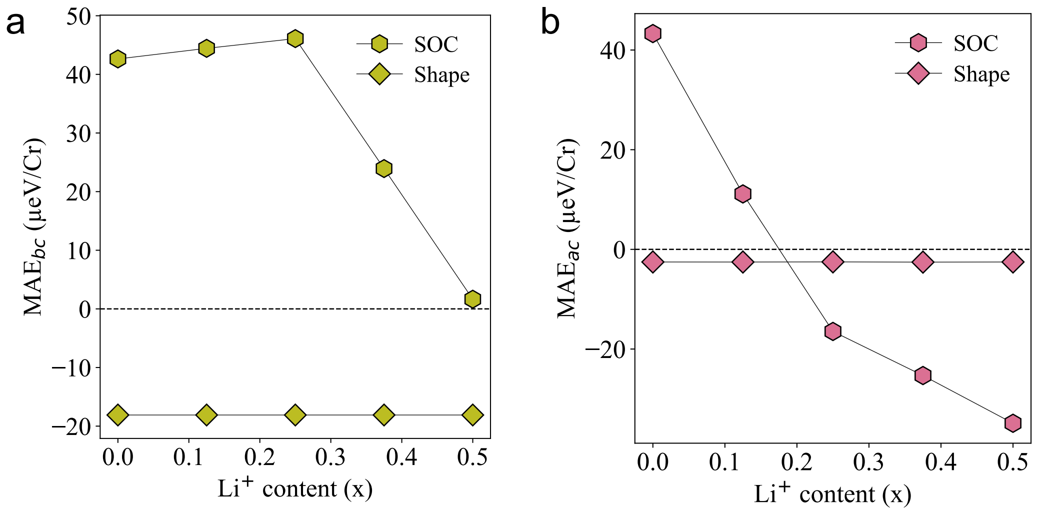

Figure S19. Evolution of the contribution of SOC and Shape anisotropy to a) MAE_{bc} and b) MAE_{ac} as a function of Li^+ content.

2.1 Electron doping effect on $CrPS_4$

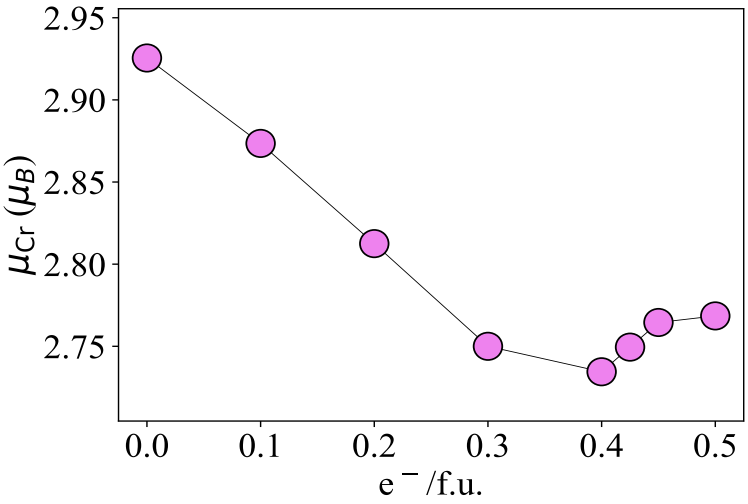

Figure S20. Evolution of magnetic moments for Cr atoms as a function of electron doping (e^-/f.u.) for U = 0.25 eV.

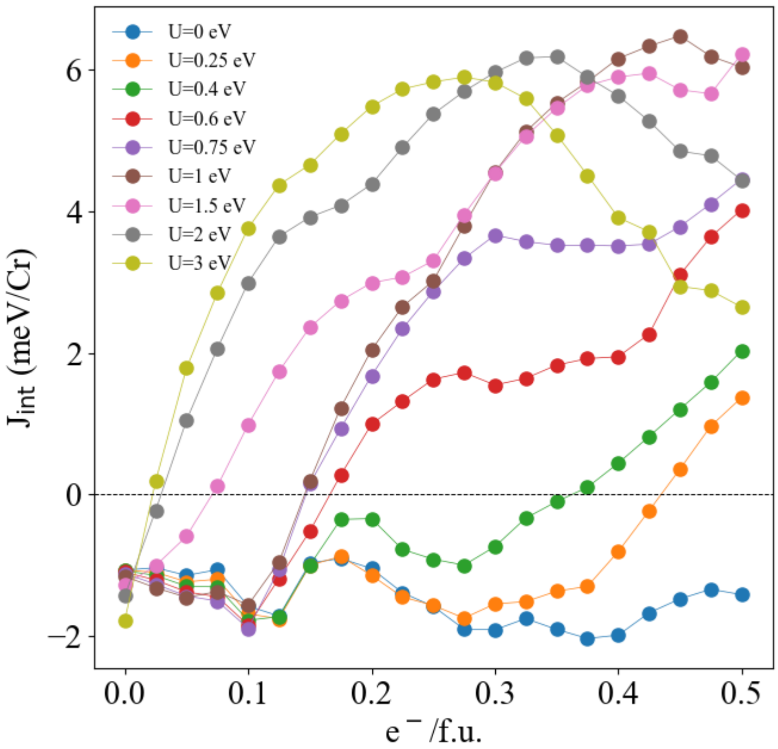

Figure S21. Evolution of J_{int} as a function of electron doping (e^-/f.u.), showing that larger U values favour an earlier stabilization of ferromagnetism.

In Figure S21 we observe that the carrier density at which the AF–FM transition occurs depends on the Hubbard U parameter, with larger U values favouring an earlier stabilization of interlayer ferromagnetism. For low U values (0 eV ≤ U < 1.5 eV), the J_{int} remains nearly constant until it reaches a saturation point in which it increases and drives an AF-to-FM transition upon occupation of the e_g orbitals. In contrast, for higher U values (1.5 eV ≤ U ≤ 3 eV), the spin up Cr e_g states are the first unoccupied conduction bands (Figure S10). Consequently, as soon as one increases the electron density, there is a continuous stabilization of interlayer ferromagnetism due to the rapid occupation of the spin up e_g orbitals.

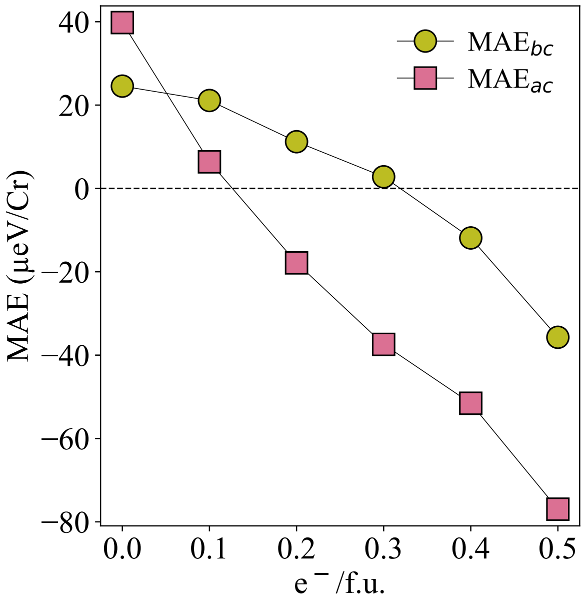

Figure S22. Evolution of MAE_{bc} and MAE_{ac} as a function of electron doping (e^-/f.u.).

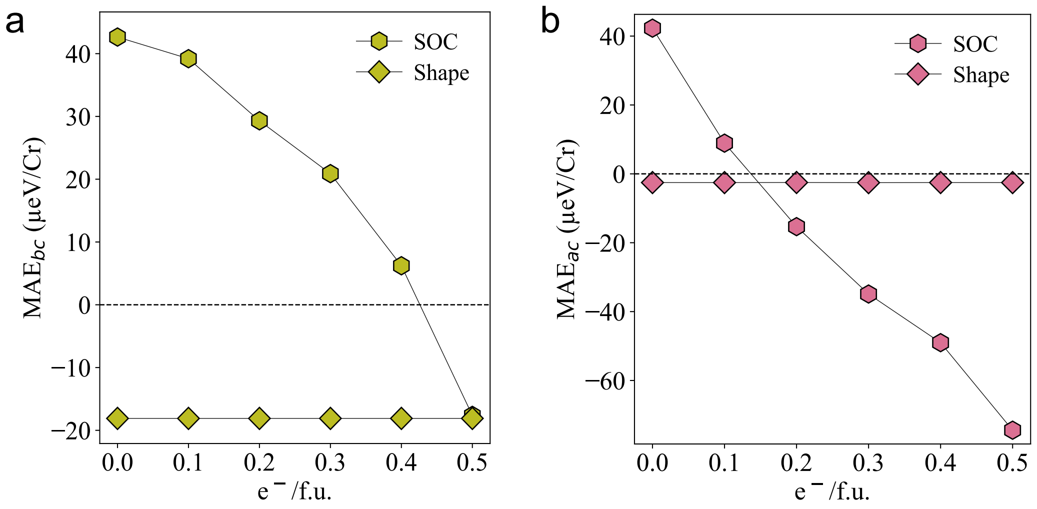

Figure S23. Evolution of the contribution of SOC and Shape anisotropy to a) MAE_{bc} and b) MAE_{ac} as a function of electron doping (e^-/f.u.).

Upon electron doping, we observe an enhancement of the magnitude of the coupling between the in-plane (d_{x2-y2}, d_{xy}) orbitals, which ultimately leads to the stabilization of in-plane magnetic anisotropy (Figure S24). This is clearly depicted in Figure S25, which illustrates the difference in the MAE (ΔMAE) between doped and pristine $CrPS_4$, showing that upon doping there is a substantial enhancement of the in-plane contribution from the (d_{x2-y2}, d_{xy}) orbitals.

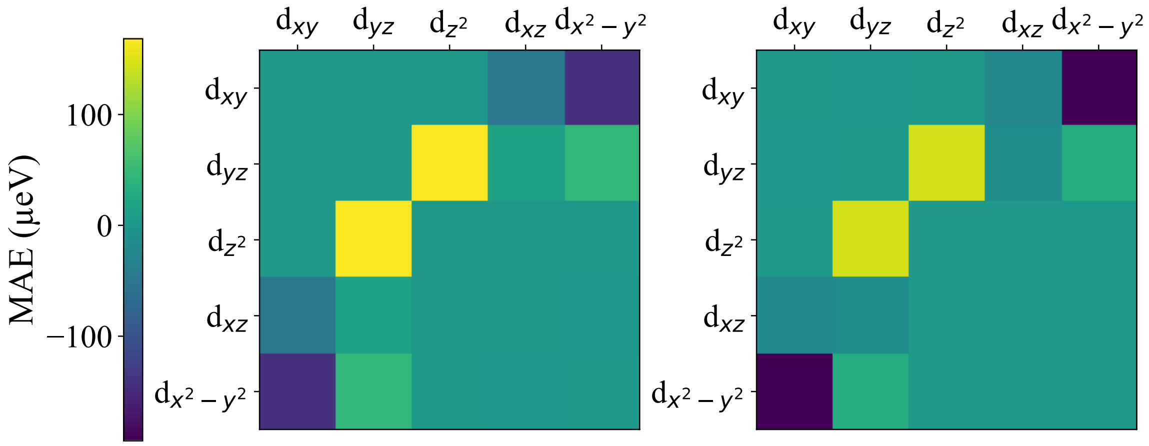

Figure S24. Orbital-resolved MAE for Cr d-orbitals in pristine $CrPS_4$ (left) and in $CrPS_4$ upon 0.5 e^- /f.u. electron doping (right).

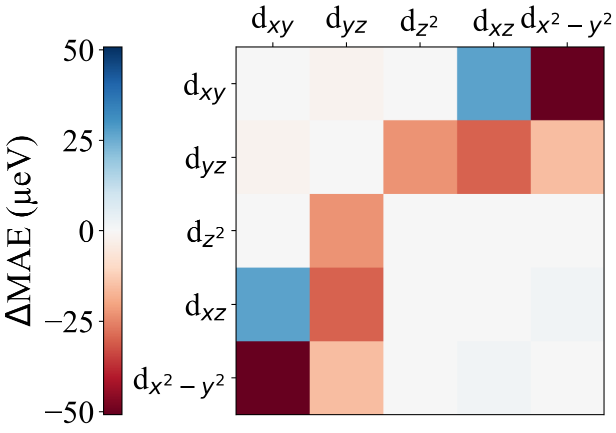

Figure S25. Difference in the MAE (ΔMAE) between doped and pristine $CrPS_4$, obtained by $\Delta MAE = MAE_{CrPS4\text{-}doped} - MAE_{CrPS4}$.

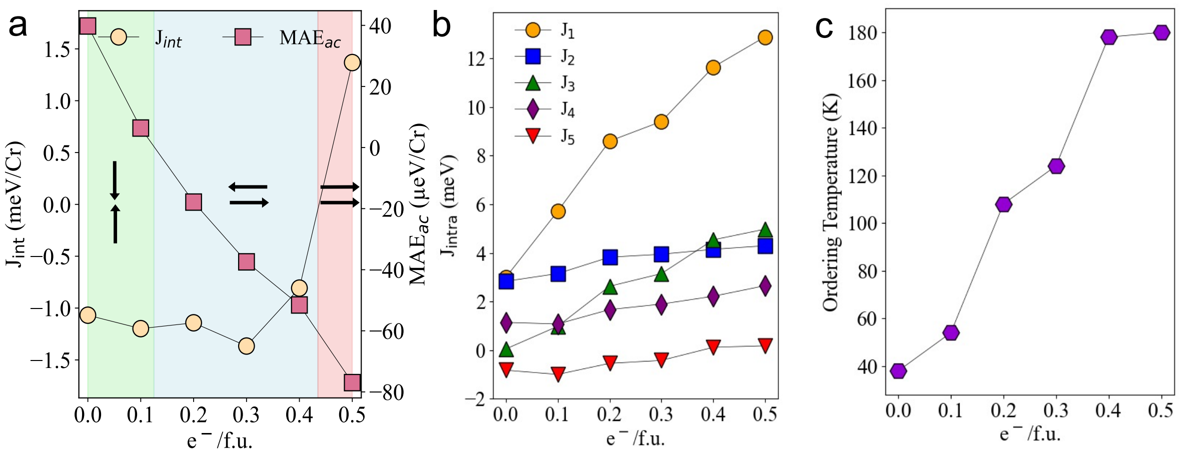

Figure S26. Evolution of the exchange interactions and ordering temperature for $CrPS_4$ as a function of electron doping. a) Evolution of interlayer exchange interaction (J_{int}) and MAE, b) intralayer couplings J_1-J_5 and c) magnetic ordering temperature as a function of electron doping (e^-/f.u.).

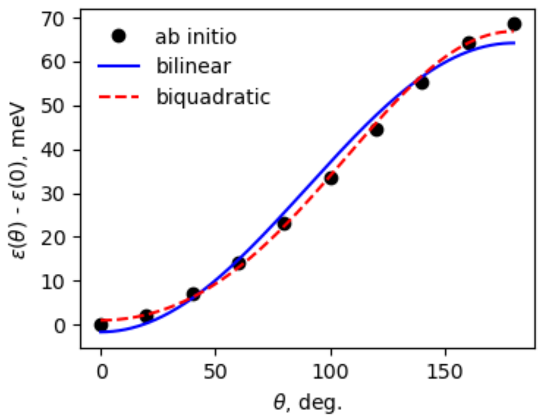

Figure S27. Total energy evolution as a function of rotation angle (θ) for metallic $CrPS_4$ along with the corresponding quadratic (red dashed line) and linear (blue solid line) fittings.

In Figure S27 we evaluate the biquadratic interactions for metallic $CrPS_4$. It shows the evolution of the total energy as a function of θ, depicting that spin rotation becomes energetically more unfavourable compared to pristine $CrPS_4$ (Figure S27 vs S7, respectively), which is attributed to the enhanced FM intralayer exchange interactions induced by electron doping (Figure S26), which in turn destabilize the AF state. Importantly, as one increases the electron doping and drives $CrPS_4$ to a metallic state, the biquadratic contribution becomes more noticeable compared to pristine $CrPS_4$ (see Figures S27 and S7). However, even for metallic $CrPS_4$ its contribution is relatively small, and the total energy evolution extracted from our calculations can be accurately fitted linearly. Note that, to simulate metallic $CrPS_4$, we employed a modest doping level of 0.5 e^-/cell, which is sufficient to induce a metallic state. Higher doping levels further strengthen the FM intralayer exchange interactions, leading to instabilities in noncollinear calculations at intermediate spin angles.

3. TBA^+ intercalated $CrPS_4$

We obtain Bader charges for pristine $CrPS_4$ and $(TBA)_{0.25}CrPS_4$ (Table S7). For the pristine compound, the analysis confirms that the net number of transferred electrons is zero, as expected for a neutral system. It also shows that charge flows from Cr and P toward S, consistent with the higher electronegativity of S and the oxidation states in $CrPS_4$ (Cr^{3+}, P^{5+} and S^{2-}). In the hybrid $(TBA)_{0.25}CrPS_4$ system, the most significant effect is that each S atom attracts more electron density than in $CrPS_4$ (1 e^- versus 0.96 e^-), which originates from electrons donated by the TBA^+ molecules. Consequently, the Cr atoms lose less electron density in the heterostructure compared to the pristine material (1.49 e^- versus 1.52 e^-).

Table S7. Bader charge transfer analysis for $CrPS_4$ and $(TBA)_{0.25}CrPS_4$ per atom. Atomic charge variations are expressed respect to the charge included in the pseudopotentials. For each compound the total transferred electrons are obtained considering that there are 4 Cr, 4 P, 16 S, 16 C, 1N and 36 H atoms. Positive and negative signs indicate the depletion and gain of electrons, respectively.

Atom	$CrPS_4$	$(TBA)_{0.25}CrPS_4$
Cr	1.5188	1.4899
P	2.3219	2.2772
S	-0.9602	-0.9983
C		0.0995
N		-1.4515
H		0.0212

In $(TBA)_{0.25}CrPS_4$, the $CrPS_4$ layers accept a total of 0.90 e^-, calculated by multiplying the charge accepted or donated by each atom by the number of atoms in the supercell $e^-_{\text{accepted, CrPS4}} = 1.4899\times4 + 2.2772\times4 - 0.9983\times16 = -0.90$ e^-. These electrons originate from the TBA^+ cations, which collectively donate the same amount $e^-_{\text{transferred, TBA}} = 0.0995\times16 - 1.4515\times1 + 0.0212\times36 = +0.90$ e^-, resulting in a charge-neutral heterostructure.

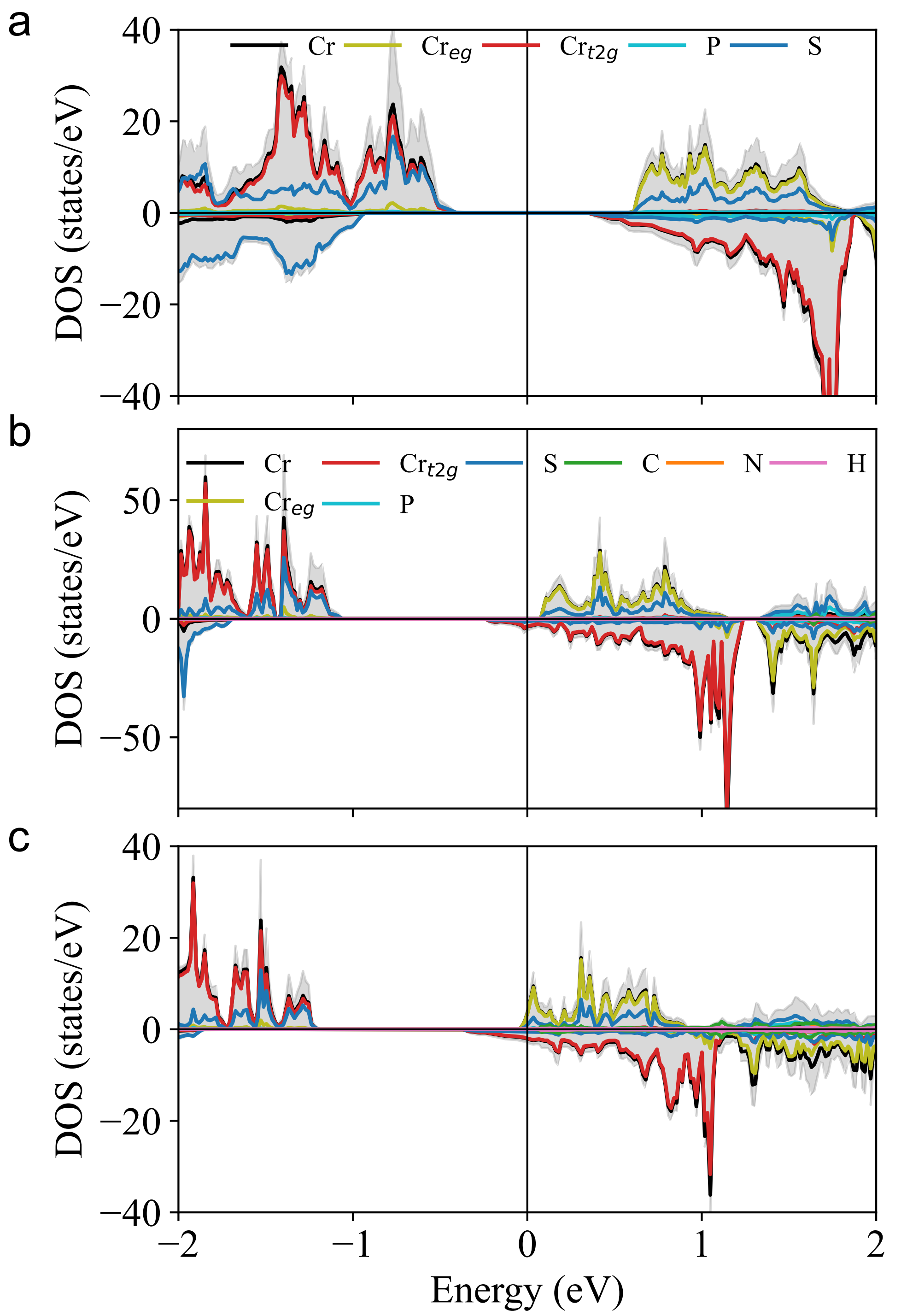

Figure S28. Orbital resolved DOS for a) $CrPS_4$, b) $(TBA)_{0.125}CrPS_4$ and c) $(TBA)_{0.25}CrPS_4$.

Table S8. Values of SOC, Shape and total MAE for $CrPS_4$, $(TBA)_{0.125}CrPS_4$ and $(TBA)_{0.25}CrPS_4$ with respect the easy *c* magnetization axis of pristine $CrPS_4$.

Material	Contribution to MAE	MAE_{bc} (μeV/Cr)	MAE_{ac} (μeV/Cr)
	SOC	42.6	42.3
$CrPS_4$	Shape	-18.1	-2.5
	Total	24.5	39.8
	SOC	20.1	-9.9
$(TBA)_{0.125}CrPS_4$	Shape	-35.4	-15.5
	Total	-15.3	-25.4
	SOC	9.6	-33.7
$(TBA)_{0.25}CrPS_4$	Shape	-31.2	-14.9
	Total	-21.6	-48.6

Table S9. Values of intralayer exchange interactions J_1-J_5 for $CrPS_4$ and for the intercalated compounds $(TBA)_{0.125}CrPS_4$ and $(TBA)_{0.25}CrPS_4$ with their corresponding values of T_N and T_C.

Material	J_1 (meV)	J_2 (meV)	J_3 (meV)	J_4 (meV)	J_5 (meV)	T_N (K)
$CrPS_4$	2.85	2.59	0.05	1.11	-0.83	39
						T_C (K)
$(TBA)_{0.125}CrPS_4$	6.25	3.55	1.17	1.18	-1.01	51
$(TBA)_{0.25}CrPS_4$	9.40	4.62	3.35	1.81	-0.24	104

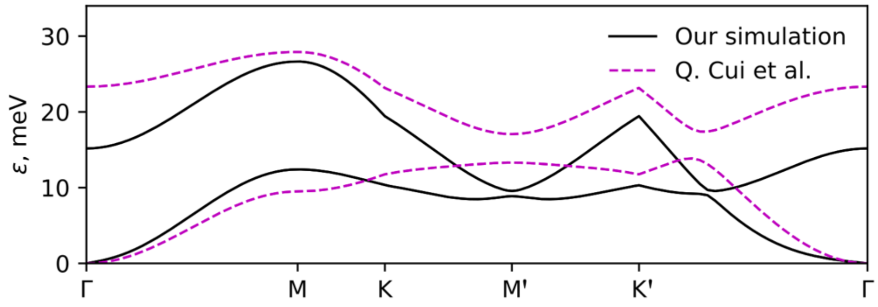

Figure S29. Comparison of the magnon dispersion calculated from our model (black solid lines) with the model of Q. Cui et al.[10]

For the calculation of magnons, we have accounted for anisotropic contributions, therefore introducing the spin Hamiltonian:

$$H = -\sum_{i\neq j} J_{ij} \vec{S}_i \cdot \vec{S}_j + \sum_i \left(\mathrm{A}_x (S_\mathrm{i}^x)^2 + \mathrm{A}_y (S_\mathrm{i}^y)^2 \right) \tag{5}$$

The parameters $\mathrm{A_x}$ and $\mathrm{A_y}$ are the anisotropy constants for $CrPS_4$, $(TBA)_{0.125}CrPS_4$, and $(TBA)_{0.25}CrPS_4$, as listed in Table S8.

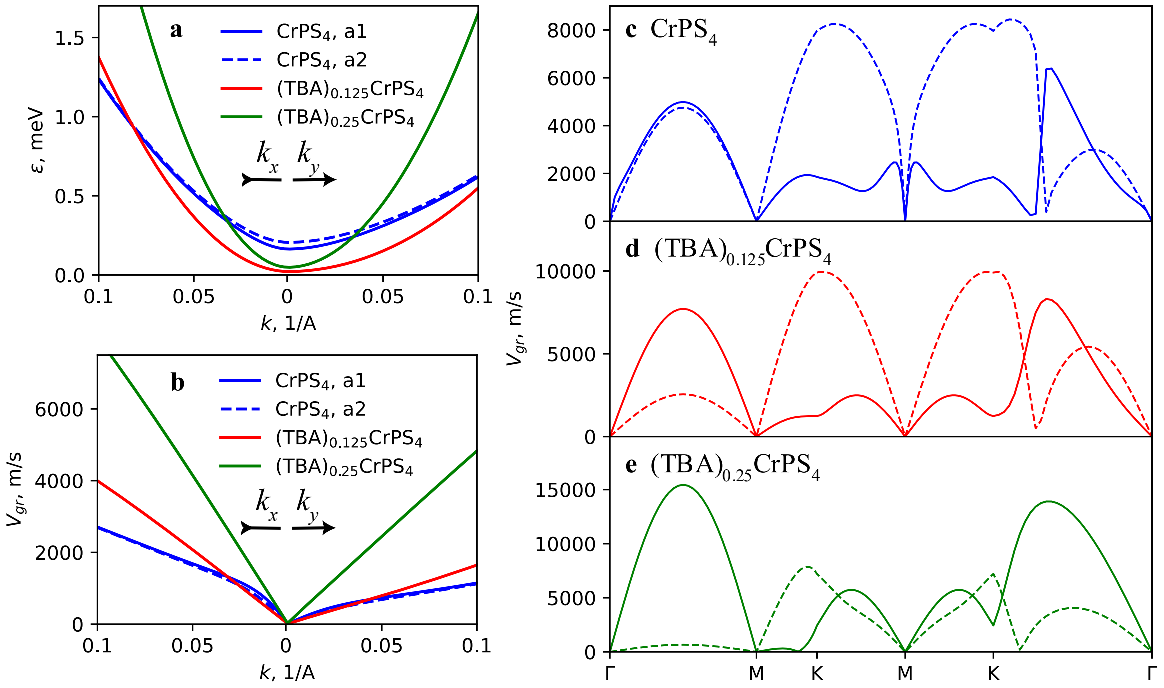

Figure S30. (a) Magnon dispersion close to Γ-point along the *x* and *y* for $CrPS_4$, $(TBA)_{0.125}CrPS_4$ and $(TBA)_{0.25}CrPS_4$. The dashed and solid lines in $CrPS_4$ refers to the non-degenerate acoustic magnons bands. (b) Group velocities close to Γ-point along the *x* and *y* directions. (c-e) Absolute values of group velocities of acoustical (solid lines) and optical (dashed lines) magnons calculated for (c) $CrPS_4$, (d) $(TBA)_{0.125}CrPS_4$ and (e) $(TBA)_{0.25}CrPS_4$.

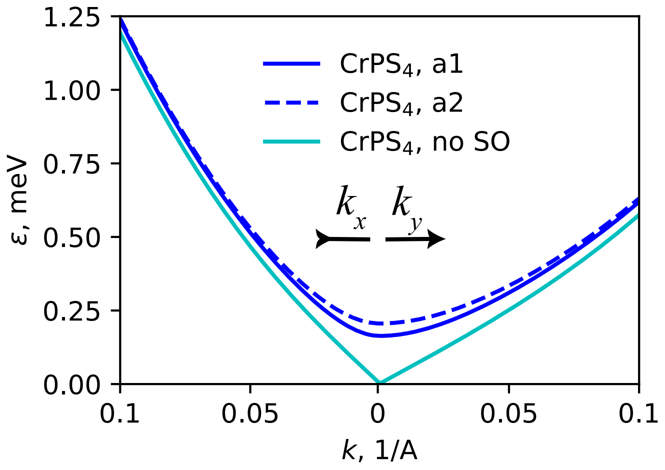

Figure S31. Magnon dispersion around Γ for $CrPS_4$ in the presence and absence of SOC.

Figure S30a shows that the anisotropy opens a magnon gap in all materials, with the largest gap occurring in $CrPS_4$ due to its antiferromagnetic order. In addition, the two low-energy magnon branches that are degenerated within the Heisenberg approximation become split by the triaxial anisotropy (Figure S31). Figure S30b illustrates how anisotropy affects the magnon group velocities, with its most pronounced effect being the suppression of the finite group velocity due to the quadratic behavior of the magnon dispersion around Γ. In Figure S30c we show the absolute values of the acoustic and optical magnon dispersions calculated along the high-symmetry path of $CrPS_4$. The x- and y- directions of the monoclinic cell correspond to Γ–M and Γ– K' branches, respectively. The presence of TBA^+ molecules enhances the magnon velocities at most k-points and makes their propagation more isotropic: upon TBA^+ intercalation, the maximum velocities along the x- and y-directions of the monoclinic cell become more similar.

REFERENCES

(1) Wu, F.; Gibertini, M.; Watanabe, K.; Taniguchi, T.; Gutiérrez-Lezama, I.; Ubrig, N.; Morpurgo, A. F. Gate-Controlled Magnetotransport and Electrostatic Modulation of Magnetism in 2D Magnetic Semiconductor $CrPS_4$. *Advanced Materials* **2023**, *35* (30), 2211653.

(2) Kartsev, A.; Augustin, M.; Evans, R. F. L.; Novoselov, K. S.; Santos, E. J. G. Biquadratic Exchange Interactions in Two-Dimensional Magnets. *NPJ Comput Mater* **2020**, *6* (1), 150.

(3) Amirabbasi, M.; Kratzer, P. Effect of Biquadratic Magnetic Exchange Interaction in the 2D Antiferromagnets MPS_3 (M = Mn, Fe, Co, Ni). *Phys Rev Mater* **2024**, *8* (8), 084005.

(4) Peng, Y.; Ding, S.; Cheng, M.; Hu, Q.; Yang, J.; Wang, F.; Xue, M.; Liu, Z.; Lin, Z.; Avdeev, M.; Hou, Y.; Yang, W.; Zheng, Y.; Yang, J. Magnetic Structure and Metamagnetic Transitions in the van Der Waals Antiferromagnet $CrPS_4$. *Advanced Materials* **2020**, *32* (28), 2001200.

(5) Sun, L.; Gibertini, M.; Scarfato, A.; Liao, M.; Wu, F.; Morpurgo, A. F.; Renner, C. Coupling between Magnetism and Band Structure in a 2D Semiconductor. *arXiv* **2025**, 2505.09946.

(6) Liu, N.; Wang, C.; Zhang, Y.; Pang, F.; Cheng, Z.; Zhang, Y.; Ji, W. Intralayer Strain Tuned Interlayer Magnetism in Bilayer CrSBr. *Phys Rev B* **2024**, *109* (21), 214422.

(7) Wang, Y.; Luo, N.; Zeng, J.; Tang, L.-M.; Chen, K.-Q. Magnetic Anisotropy and Electric Field Induced Magnetic Phase Transition in the van Der Waals Antiferromagnet CrSBr. *Phys Rev B* **2023**, *108* (5), 054401.

(8) Ye, C.; Wang, C.; Wu, Q.; Liu, S.; Zhou, J.; Wang, G.; Söll, A.; Sofer, Z.; Yue, M.; Liu, X.; Tian, M.; Xiong, Q.; Ji, W.; Renshaw Wang, X. Layer-Dependent Interlayer Antiferromagnetic Spin Reorientation in Air-Stable Semiconductor CrSBr. *ACS Nano* **2022**, *16* (8), 11876–11883.

(9) Henríquez-Guerra, E.; Ruiz, A. M.; Galbiati, M.; Cortés-Flores, Á.; Brown, D.; Zamora-Amo, E.; Almonte, L.; Shumilin, A.; Salvador-Sánchez, J.; Pérez-Rodríguez, A.; Orue, I.; Cantarero, A.; Castellanos-Gomez, A.; Mompeán, F.; Garcia-Hernandez, M.; Navarro-Moratalla, E.; Diez, E.; Amado, M.; Baldoví, J. J.; Calvo, M. R. Strain Engineering of Magnetoresistance and Magnetic Anisotropy in CrSBr. *Advanced Materials* **2025**, 2506695.

(10) Cui, Q.; Bai, X.; Delin, A. Anisotropic Magnon Transport in Van Der Waals Ferromagnetic Insulators. *Adv Funct Mater* **2025**, *35* (1), 2407469.
